# A unified evaluation framework for revising inappropriate arguments with edit-level guidance

**DOI:** 10.3389/frai.2026.1869755

**Published:** 2026-10-30

**Authors:** Timon Ziegenbein, Sonja Lukic, Henning Wachsmuth

**Affiliations:** Faculty of Electrical Engineering and Computer Science, Institute of Artificial Intelligence, Leibniz University Hannover, Hannover, Germany

**Keywords:** computational argumentation, large language models, public deliberation, reinforcement learning, span-level text editing

## Abstract

In an appropriately-phrased argument, the language supports credibility and emotional engagement while remaining proportional to the issue at hand. Arguments that fall short of these properties undermine public deliberation and the quality of democratic discourse, motivating research on their automatic revision. Because ex-post moderation is costly and time-consuming, suggesting revisions at creation time is a promising alternative. We unify two prior approaches to revising inappropriate arguments under a common edit-operation framework: a PPO-based approach that aligns a large language model (LLM) to generate full rewrites from which edit suggestions are extracted ex-post using a diff tool, and a GRPO-based approach that makes the LLM generate self-contained span-level edit suggestions directly. The framework defines the desired properties of the task and introduces two additional quality criteria: pattern conformity at the argument level and appropriateness at the edit level. Placing both paradigms on a common footing yields an approach-agnostic basis for evaluating edit suggestions, independent of whether they are generated directly or derived from a full rewrite. The results of a moderator-style user study, in which human moderators iteratively selected and applied edit suggestions from our approaches, suggest that overall revision quality is high (79.2% of arguments revised to full appropriateness), while also revealing span-level shortcomings in rewriting-derived edit suggestions that motivate the quality criteria introduced here. A comprehensive evaluation of eight argument revision approaches reveals that approaches generating edit suggestions directly and optimizing jointly over multiple quality criteria achieve human-likeness competitive with large prompted models, while approaches optimizing for appropriateness alone systematically produce edit suggestions that violate structural quality requirements. Across both studies, the two generation paradigms emerge as failing in different ways: edit suggestions extracted from full rewrites are frequently interdependent and lose fluency when applied selectively, whereas edit suggestions generated directly are self-contained but individually less effective and, hence, need iterative application. Together, these contributions establish argument revision via span-level edit suggestions as a unified and interpretable task, with direct implications for human-in-the-loop moderation and AI-assisted deliberation.

## 1 Introduction

Online platforms facilitate public deliberation at unprecedented scale, enabling individuals to engage with controversial topics across political, social, and ethical domains. Argumentation is a central mechanism of such deliberation—participants exchange reasons, challenge positions, and respond to counterarguments. Research on computational argumentation spans argument mining (Stab and Gurevych, 2017; Cabrio and Villata, 2018; Lawrence and Reed, 2019), argument quality assessment (Habernal and Gurevych, 2016; Wachsmuth et al., 2017; Toledo et al., 2019), and argument generation (Hua and Wang, 2018; Syed et al., 2021; Skitalinskaya et al., 2023). Among the dimensions addressed in argument quality assessment, a fundamental precondition for productive deliberation is that arguments are phrased in an *appropriate* manner (Wachsmuth et al., 2017): arguments that violate this by being hostile, incoherent, or disengaged from the discourse context obstruct genuine exchange and undermine the epistemic function of argumentation (Habermas, 1979, 1984, 1990). Wachsmuth and Werner (2020) are the first to generally assess appropriateness as an argument quality dimension computationally; Ziegenbein et al. (2023) model it more specifically through the absence of reasons for inappropriateness, operationalized in a taxonomy that covers 14 dimensions related to toxic emotions, missing commitment, missing intelligibility, and other issues. Unlike binary toxicity classifiers (Wulczyn et al., 2017; Borkan et al., 2019) or offensive-language detectors (Davidson et al., 2017; He et al., 2023), this taxonomy is multi-label and argumentation-specific: properties that make it better suited to the nuanced demands of improving deliberation quality, as it covers not only toxic language but also discourse-level violations such as intelligibility issues or missing commitment to a debate.

Platforms currently address inappropriate content predominantly through largely manual ex-post moderation: detecting and removing contributions after publication (Chandrasekharan et al., 2018; Gorwa et al., 2020). Existing ex-ante approaches (i.e., that intervene before publication), similarly focus on preventing submission rather than guiding users toward appropriate reformulation (Gorwa et al., 2020). Recent work has begun to address this limitation: platform-deployed nudges prompt authors to reconsider their phrasing before posting (Katsaros et al., 2022; Singhal et al., 2023); others explain which community rules are violated (Horta Ribeiro et al., 2025); and yet others go furthest by suggesting or directly generating revised versions of toxic text (Laugier et al., 2021; Logacheva et al., 2022; Hallinan et al., 2023). However, these approaches lack specificity: nudges and rule explanations identify problems without suggesting how to address them, while full rewrites do not make clear which specific spans were changed or why. A more informative ex-ante alternative is to provide targeted revision guidance at creation time, explaining what needs to change rather than issuing a simple warning or generating a full rewrite. *Edit-based approaches* seem particularly promising for these purposes: rather than rewriting the full text, they operate on specific spans to produce localized, interpretable changes (Du et al., 2022; Raheja et al., 2023), in contrast to automatic rewriters that regenerate long text passages entirely (Shu et al., 2024). First research in the domain of toxic language explores targeting and replacing offensive spans (Wu et al., 2019; Dale et al., 2021), with counterfactual generation offering an additional explanatory signal (Bhan et al., 2026). Li et al. (2025) propose a tool that pairs explainable detection with personalized modification suggestions, and find that users indeed expect support beyond detection alone. Recent work continues to advance the detection of harmful online language, including cyberbullying and cyberstalking (Vikas et al., 2025) as well as multimodal detection combining textual and visual content (Sreeraj et al., 2025). At the same time, generative assistance is not necessarily beneficial: in a controlled social-media experiment, Møller et al. (2026) observe that AI interventions such as feedback on drafts and reply suggestions can raise engagement while lowering the perceived quality and authenticity of discussion, which motivates keeping the human in control of which suggestions are adopted. The value of making the edit itself the unit of optimization is also visible outside argumentation: in grammatical error correction, weighting rewards toward edited tokens rather than whole sequences yields more targeted, minimal corrections (Liang et al., 2025; Li and Wan, 2026). However, inappropriate argumentation is not reducible to toxic language or grammatical error correction: it additionally encompasses missing intelligibility and missing commitment (Ziegenbein et al., 2023).

In two prior works, we contribute toward closing this gap: Ziegenbein et al. (2024) train a proximal policy optimization (PPO) (Schulman et al., 2017) model to optimize the behavior of a large language model (LLM) to generate full rewrites of inappropriate arguments, using an argument-level reward combining two quality criteria: appropriateness and content preservation. Following the reinforcement learning from human feedback paradigm (Christiano et al., 2017; Stiennon et al., 2020; Ouyang et al., 2022), this approach avoids the need for large pairwise preference corpora, which are costly to collect and often require expert annotators, by treating pretrained classifiers as reward proxies. Edit suggestions are not an explicit output: they must be extracted ex-post via textual differencing, as done by Ziegenbein et al. (2026) for evaluation purposes. It is not a priori clear, however, whether such diff-extracted edit suggestions are well-targeted at the span level (in the article at hand, we examine this empirically through a moderator-style user study in which human moderators select among edit suggestions to revise inappropriate arguments). In particular, Ziegenbein et al. (2026) train a group relative policy optimization (GRPO) (Shao et al., 2024) model to make an LLM generate self-contained span-level edit suggestions directly in human-like ways. To this end, they propose edit-level rewards for fluency, conformity to typical human editing patterns, and content preservation, alongside an argument-level appropriateness reward.

Building on Ziegenbein et al. (2024) and Ziegenbein et al. (2026), we generalize the revision of inappropriate arguments via editing into a unified, approach-agnostic framework. Specifically, our two prior approaches differ in granularity: one operates at the argument level, the other at the span level. We unify them under a shared vocabulary of span-level edit operations, with rewriting-derived edit suggestions extracted ex-post using a diff tool, which enables joint analysis across paradigms. Within this framework, we collect the quality criteria used across prior work for model training and evaluation, and expand them with two additional criteria. At the argument level, we introduce a conformity criterion measuring whether the revision follows recognizable human editing patterns. At the edit level, we introduce an appropriateness criterion linking each span-level change to the dimension of inappropriateness it addresses. This unification makes it possible to compare any approach that produces edit suggestions—regardless of its underlying paradigm—with respect to revision strengths and weaknesses, and to analyze how different approaches prioritize the dimensions of inappropriateness. Figure 1 illustrates the targeted human-in-the-loop revision process, in which a human moderator iteratively selects and applies AI-generated edit suggestions to produce a revised argument.

**Figure 1:**
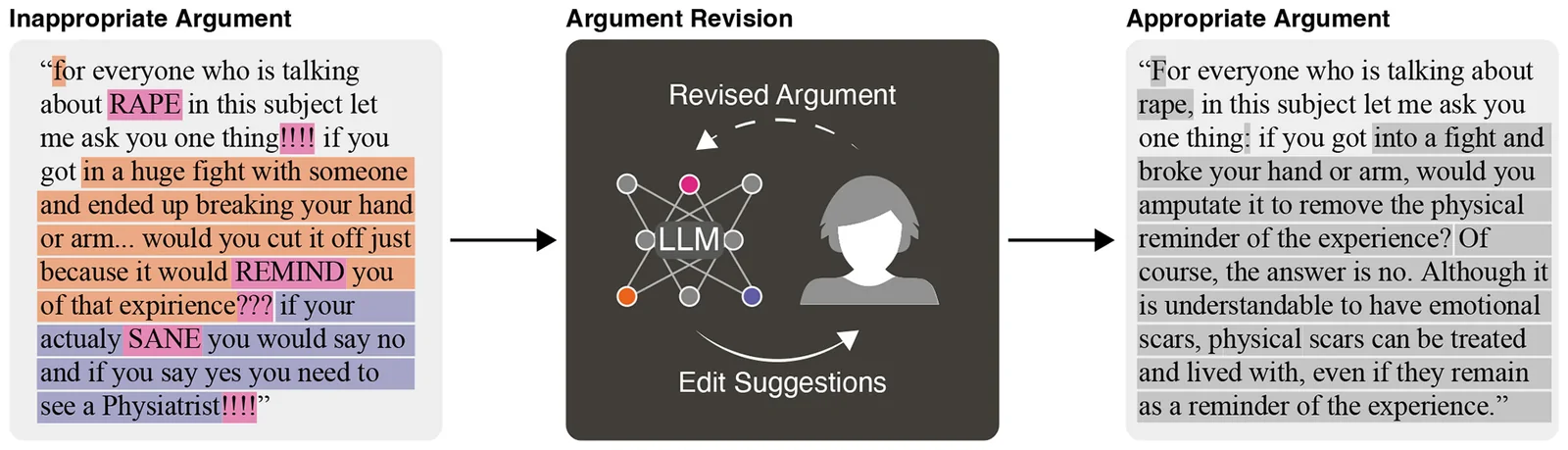
Human-in-the-loop argument revision process: AI approaches automatically generate span-level edit suggestions for an inappropriate argument; a human moderator iteratively selects and applies edit suggestions in collaboration with the AI to produce a revised argument. The focus of our proposed framework is the computational generation of edit suggestions.

Beyond revisiting our two foundational prior works, this paper makes four original contributions:

1. We provide a unified, approach-agnostic formalism for the revision of inappropriate arguments via editing, defining the desired properties of the task, so that any approach producing edit suggestions can be assessed on a common basis irrespective of how its suggestions have been generated.
2. We conduct a moderator-style user study and provide evidence that rewriting-derived, diff-extracted edit suggestions achieve effective argument-level revision, while revealing edit-level shortcomings that motivate the need for edit suggestion-aware quality criteria.
3. We introduce two further quality criteria—pattern conformity at the argument level and appropriateness at the edit level—that enable fine-grained assessment and attribution of argument revisions.
4. We apply the unified framework to analyze eight existing argument revision approaches, characterizing their revision behavior and how they prioritize the different dimensions of inappropriateness.

## 2 Unified framework

This section introduces the unified framework for argument revision via edit suggestions. We first formalize the edit operation vocabulary and define the quality criteria that govern both individual edit suggestions and revised arguments as a whole. The space of argument revision approaches is then characterized along three dimensions: training paradigm, output type, and granularity.

### 2.1 Argument revision via edit suggestions

Throughout, we use the term *edit suggestion* to refer to a span-level change proposed by an approach for application to an argument, and *revision* to refer to the resulting revised argument obtained by applying a set of edit suggestions. Approaches for inappropriate argument revision via edit suggestions share a common goal—making arguments more appropriate—but may instantiate it through fundamentally different mechanisms. A rewriting approach generates a full revision of the argument and recovers edit suggestions ex-post; an editing approach generates self-contained span-level edit suggestions directly. Despite this difference in generation behavior, all approaches produce outputs expressible in a common vocabulary of span-level edit operations, illustrated in Figure 1. We formalize the vocabulary of edit operations and the quality criteria that govern it as follows.

#### 2.1.1 Edit operation formalization

Let *a* denote an argument to be revised. A *span-level edit suggestion* is a pair *e*: = (*s, t*) consisting of a source span *s* and a target span *t* in *a*. Three edit suggestion types arise from this formalism: a *replacement* substitutes a non-empty source span with a non-empty target span (*s*≠ϵ, *t*≠ϵ); a *deletion* removes a source span without substitution (*t* = ϵ); and an *addition* inserts a target span at a position in *a* without removing existing content (*s* = ϵ).

An *edit suggestion set* *E* = {*e*_1_, …, *e*_*k*_} collects all edit suggestions for a given argument. We write *a*′ = apply(*a, E*) for the revised argument obtained by applying all edit suggestions in *E*; since edit suggestions operate on non-overlapping spans, the result is order-independent. The revision goal is to find an edit suggestion set E^*^ such that apply(a, E^*^) is free of reasons for inappropriateness (Ziegenbein et al., 2023), with as few and as minimal changes to the original argument as possible, reflecting the inherent tension between improving appropriateness and preserving the original text (Ziegenbein et al., 2024). Let *A* denote the set of all arguments and **E** the set of all possible edit suggestion sets. We define an *argument revision approach* as a function π:*A*→**E** that maps each argument to an edit suggestion set.

#### 2.1.2 Argument-level quality criteria

We define four criteria for assessing the quality of a revised argument *a*′ = apply(*a, E*) as a whole, abbreviated as Sim (Similarity), Flu (Fluency), Con (Conformity), and App (Appropriateness), subscripted with *a* for argument-level and *e* for edit-level. The criteria consolidate evaluation dimensions from prior work (Ziegenbein et al., 2024, 2026) by examining both argument and edit levels jointly: where a criterion existed at only one level, we considered whether a meaningful analog at the other level was warranted, which motivated introducing Con_*a*_ as a novel argument-level criterion.

- ${\text{Sim}}_{a}\left(a,a^{\prime }\right)$: the extent to which the revision preserves the semantic content of the original argument.
- ${\text{Flu}}_{a}\left(a^{\prime }\right)$: the extent to which the revised argument is fluent.
- Con_*a*_(*a, E*): the extent to which *E* reflects typical human revision strategies.
- ${\text{App}}_{a}\left(a^{\prime }\right)$: the extent to which the revised argument is appropriate—the primary revision goal.

#### 2.1.3 Edit-level quality criteria

We define four criteria for assessing an individual edit suggestion *e* in the context of argument *a*, applying the same cross-level reasoning:

- Sim_*e*_(*e, a*): the extent to which the edit suggestion preserves the semantic content of the text it modifies.
- Flu_*e*_(*e, a*): the extent to which the edit suggestion preserves the fluency of the argument.
- Con_*e*_(*e, a*): the extent to which the edit suggestion conforms to typical human editing patterns.
- App_*e*_(*e, a*): the extent to which the edit suggestion increases the appropriateness of *a*.

The first three—introduced by Ziegenbein et al. (2026)—mirror the argument-level criteria above, but are assessed locally at the edit level. Specifically, Con_*e*_ has an argument-level analog in Con_*a*_: while Con_*e*_ assesses whether a single edit suggestion's operations conform to human editing patterns, Con_*a*_ captures whether the full edit suggestion set *E*, considered jointly, conforms to human revision patterns at the document level. Together they define *human-likeness* as a function of the three criteria, as given in Equation 1:


$$\begin{array}{l} \text{HL}\left(e,a\right):=f\left({\text{Sim}}_{e}\left(e,a\right),{\text{Flu}}_{e}\left(e,a\right),{\text{Con}}_{e}\left(e,a\right)\right) \end{array}$$ (1)

where *f* is an aggregation function; the specific instantiation used in this work is described in Section 6. In the evaluation (Section 7.1), we extend the filter to also require App_*e*_, yielding HL_App_(*e, a*): = HL(*e, a*)∧App_*e*_(*e, a*), so that only edit suggestions that are both structurally human-like and appropriateness-improving pass the filter. These criteria are local, ensuring that each edit suggestion can be evaluated and applied independently of others—n contrast to edit suggestions derived from document-level rewrites, where applying one edit suggestion may alter the context in which another was designed to operate.

App_*e*_, by contrast, is appropriateness-specific: it assesses whether a given edit suggestion increases the appropriateness of the argument, rather than whether the edit suggestion is well-formed.

### 2.2 Edit suggestion generation paradigms

Approaches to argument revision via edit suggestions, described in detail in Section 3, can be characterized along three dimensions: the *training paradigm*, the *output type*, and the *input scope*.

#### 2.2.1 Training paradigm

The training paradigm determines how model parameters are adapted to the revision task. *Prompting* requires no task-specific training; a pre-trained LLM generates revisions from natural-language instructions without labeled data of any kind. *Reinforcement learning* fine-tunes a model via a scalar reward that encodes desired revision properties, derived from human preference annotations or from pre-trained classifiers, without requiring labeled edit suggestion pairs. *Supervised learning* trains a model directly on human-annotated (*a, E*) pairs, providing precise edit-level guidance at the cost of requiring labeled training data.

#### 2.2.2 Output type

The output type distinguishes how edit suggestions are produced. A *rewriting* approach generates a full revised argument *a*′; the edit suggestion set *E* is then derived ex-post by applying a diff algorithm to the pair (*a, a*′). Throughout this paper, we use latexdiff^1^ for this step, regardless of whether the rewriting approach is reinforcement-learned, prompting-based, or supervised. A *direct editing* approach generates the edit suggestion set *E* explicitly as a set of span-level (*s, t*) pairs, without producing a full revised argument.

#### 2.2.3 Input scope

The input scope determines how much of the argument the model receives per generation step. This is consequential: argument-level models can make globally informed revision decisions, whereas sentence-level models may miss cross-sentence dependencies relevant to consistency or strategic intervention. An *argument-level* approach processes the full argument in a single forward pass. A *sentence-level* approach processes one sentence at a time, operating over individual sentences rather than the full argument.

## 3 Approaches

In our experiments on appropriateness revision, we evaluate two prototypical implementations of the proposed framework along with various baselines. In total, these approaches span three training paradigms and two output types, as described in Section 2.2. Table 1 provides an overview; Figure 2 illustrates the contrast between the two output types; the following subsections detail all approaches.

**Table 1:** Overview of all approaches evaluated in this paper, including their training paradigm, output type, granularity level, parameter count, task training data, and source.

Approach	Training	Output	Level	Params	Task data	Source
PPO_*_	RL	Rewrite	Argument	7B	App. Corpus	Ziegenbein et al., 2024
GRPO_*_	RL	Edit pairs	Argument	8B	App. Corpus	Ziegenbein et al., 2026
Alpaca	Prompting	Rewrite	Argument	7B	–	Taori et al., 2023
Llama	Prompting	Edit pairs	Argument	8B	–	Grattafiori et al., 2024
Gemini 2.5	Prompting	Edit pairs	Argument	–	–	Comanici et al., 2025
GPT-5	Prompting	Edit pairs	Argument	–	–	–
DeLIteraTeR	Supervised	Edit pairs	Sent. & Arg.	1B	Text editing	Kim et al., 2022
CoEdIT	Supervised	Rewrite	Sentence	11B	Text editing	Raheja et al., 2023

PPO_*_ and GRPO_*_ denote the respective variant families; see Sections 3.1 and 3.2 for individual variants.

**Figure 2:**
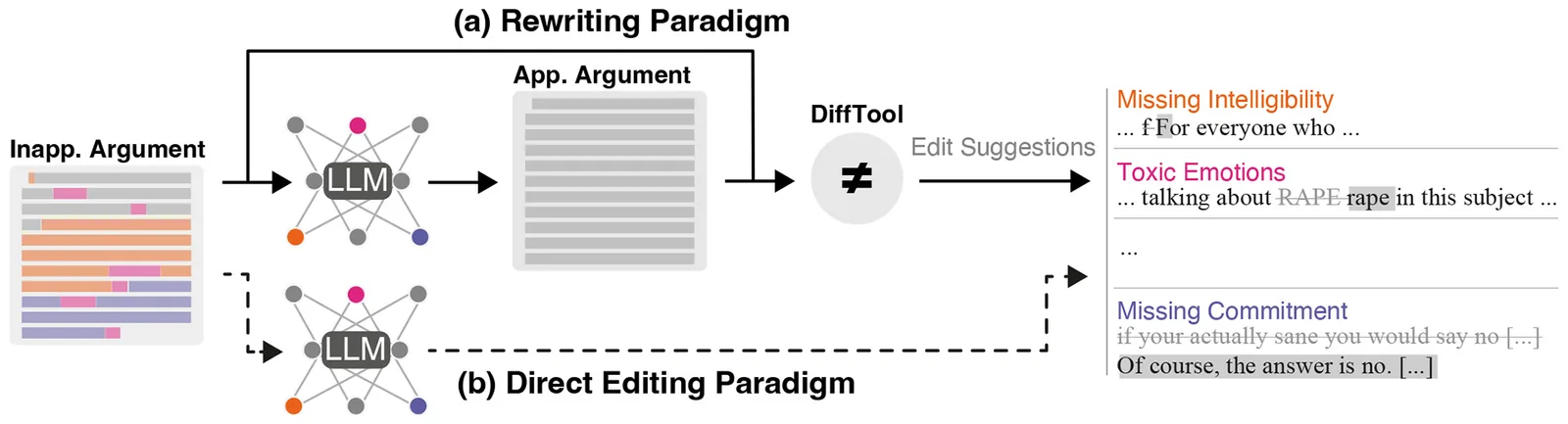
Two output types for generating span-level edit suggestions. The **(a)** *rewriting* output type (solid lines) generates a full revised argument and extracts edit suggestions ex-post via a diff tool; the **(b)** *direct editing* output type (dotted line) generates edit suggestions directly. Both output types produce edit suggestions expressed in a shared vocabulary of span-level edit operations.

### 3.1 Inappropriateness rewriting

The rewriting approach originally proposed by Ziegenbein et al. (2024) learns a policy π^*RL*^:*A*→*A* such that each output argument *a*′ = π^*RL*^(*a*)∈*A* is more appropriate than the input argument *a*∈*A* while preserving its content. Here, *a*′ is generated by π^*RL*^ first, and the associated edit suggestion set *E* is derived from (*a, a*′) ex-post. The policy is trained with PPO (Schulman et al., 2017) and LoRA (Hu et al., 2022), initializing from Alpaca (Taori et al., 2023)—an instruction-finetuned LLaMA model—using a reward that combines the argument-level criteria Sim_*a*_ and App_*a*_, as defined in Equation 2:


$$\begin{array}{l} R\left(a,a^{\prime }\right):=\alpha \cdot {\text{App}}_{a}\left(a^{\prime }\right)+\left(1-\alpha \right)\cdot {\text{Sim}}_{a}\left(a,a^{\prime }\right) \end{array}$$ (2)

where α∈[0, 1] controls the trade-off between content preservation and appropriateness improvement. π^*RL*^ is trained on the Extended Appropriateness Corpus and evaluated on the test split of the Appropriateness Corpus (both of which we summarize in Section 4). Here, App_*a*_ is realized as the appropriateness classifier of Ziegenbein et al. (2023) and Sim_*a*_ as BERTScore (Zhang et al., 2020); see Section 6 for details.

Ziegenbein et al. (2024) obtain four PPO-trained policies by varying α in *R*:

- PPO_*app*_ (α = 1.0): Only appropriateness is explicitly optimized.
- PPO_*app*>*sim*_ (α = 0.6): Appropriateness is weighted higher than similarity during optimization.
- PPO_*app* = *sim*_ (α = 0.5): Appropriateness and similarity are weighted equally.
- PPO_*app*<*sim*_ (α = 0.4): Similarity is weighted higher than appropriateness.

To derive an edit suggestion set *E* from each rewrite for analysis below, we apply latexdiff^2^ to each pair (*a, a*′), extracting span-level Replace, Delete, and Add operations (*s, t*). Crucially, the reward *R* operates entirely at the argument level: edit suggestion quality is never directly optimized, and the structure of the resulting edit suggestions is an artifact of the diff procedure rather than a training objective.

### 3.2 Inappropriateness editing

The editing approach originally proposed by Ziegenbein et al. (2026) addresses a fundamental limitation of rewriting-derived edit suggestions: edit suggestions extracted ex-post via latexdiff are artifacts of a diff procedure applied to document-level rewrites, and they frequently exhibit patterns absent from human editing— such as retaining large spans with only a single token modified. To avoid this, the editing approach trains a policy π^*RL*^:*A*→**E** that generates self-contained span-level edit suggestions directly, making human-likeness an explicit training signal.

Ziegenbein et al. (2026) instantiate the policy as Llama-3.1-8B-Instruct (Grattafiori et al., 2024), fine-tuned with GRPO (Shao et al., 2024) and LoRA (Hu et al., 2022) on the Extended Appropriateness Corpus and evaluated on the test split of the Appropriateness Corpus (Section 4). The model generates the edit suggestion set *E* as structured JSON output, prompted with category explanations and an in-context example. The reward combines two components. Let HL(*e, a*) be the human-likeness indicator from Section 2.1.3. Let *E*_HL_⊆*E* denote the subset of human-like edit suggestions and $a^{\prime }=\text{apply}\left(a,E_{\text{HL}}\right)$. Then, the two reward components are defined in Equation 3:


$$\begin{array}{l} r_{\text{edit}}\left(a,E\right):=\frac{1}{\left|E\right|}\underset{e\in E}{\sum }\text{HL}\left(e,a\right)\;r_{\text{arg}}\left(a,E\right):={\text{App}}_{a}\left(a^{\prime }\right)\cdot \text{𝕀}\left(|E_{\text{HL}}|>0\right) \end{array}$$ (3)

An edit suggestion is thus considered human-like if it is sufficiently similar to the original span, preserves fluency, and exhibits human editing patterns. Concretely, HL is realized via binary classifiers for Sim_*e*_, Flu_*e*_, and Con_*e*_ (EmbeddingGemma-300M, ModernBERT, and a decoder LM, respectively), and App_*a*_ as the appropriateness classifier of Ziegenbein et al. (2023); see Section 6 for details.

The combined reward (Equation 4) traa a factor α, set to 0.5 in Ziegenbein et al. (2026), equally weighting argument-level and edit-level objectives:


$$\begin{array}{l} R\left(a,E\right):=\alpha \cdot r_{\text{arg}}\left(a,E\right)+\left(1-\alpha \right)\cdot r_{\text{edit}}\left(a,E\right) \end{array}$$ (4)

We evaluate two variants proposed by Ziegenbein et al. (2026): GRPO_HL_, which uses the full combined reward with all three edit-level classifiers (Sim_*e*_, Flu_*e*_, Con_*e*_), and GRPO_app_, which uses argument-level reward only, without any edit-level signal. Crucially, unlike the rewriting approach, edit suggestion quality is an explicit training objective: the argument-level reward is applied only to the human-like subset *E*_HL_, directly trading off argument-level appropriateness against edit-level quality.

### 3.3 Prompting baselines

To assess the effectiveness of the two outlined framework implementations, we compare them to four prompting approaches that require no task-specific training: a pre-trained LLM is instructed to revise an inappropriate argument via natural language instructions, without any training:

- *Alpaca* (Taori et al., 2023): the instruction-finetuned Llama model used as the initialization point for the inappropriateness rewriting approach, prompted to generate a full revised argument; edit suggestions extracted ex-post via latexdiff.
- *Llama 3.1* (Grattafiori et al., 2024): Llama-3.1-8B-Instruct prompted with the same structured JSON template as the inappropriateness editing approach (Ziegenbein et al., 2026), but without GRPO fine-tuning.
- *Gemini 2.5* (Comanici et al., 2025): prompted with the same JSON template; edit suggestions are parsed directly from the output.
- *GPT-5*: prompted with the same JSON template; edit suggestions are parsed directly from the output.

Alpaca and Llama 3.1 allow us to isolate the contribution of the reinforcement learning of the two approaches; Gemini 2.5 and GPT-5 represent state-of-the-art closed-source LLMs. The *GPT-5* and *Gemini 2.5* edit suggestions follow the generation setup of Ziegenbein et al. (2026).

### 3.4 Supervised edit suggestion generation

Finally, we include supervised edit suggestion generation approaches from related work. These approaches are trained directly on human-annotated data, being provided explicit edit-level guidance at the cost of requiring labeled training data. We include two such baselines, representing contrasting designs within this paradigm:

DeLIteraTeR (Kim et al., 2022) addresses text editing as a detect-then-generate problem. A span detection component first identifies spans to be revised in the source sentence; a targeted generation component then produces the replacement for each detected span. As both the source span and the target span are produced explicitly, DeLIteraTeR generates edit suggestions (*s, t*) directly, without requiring ex-post diff extraction. The system is trained on an extension of the IteraTeR dataset (Du et al., 2022) (summarized in Section 4) with additional data from grammatical error correction, text simplification, and style transfer—tasks not directly targeting inappropriateness but covering related dimensions such as fluency, clarity, and style.

CoEdIT (Raheja et al., 2023) addresses text editing as a sequence-to-sequence problem: given an instruction and a source sentence, the model generates a full revised sentence directly. We use the largest available variant (11B parameters), fine-tuned on instruction–edit suggestion pairs covering tasks such as fluency improvement, coherence, clarity, paraphrasing, grammatical error correction, and simplification; edit suggestions are recovered ex-post via latexdiff. While not trained on inappropriateness data, CoEdIT can be applied to the task via instruction prompting, using the instruction “Make this more appropriate:” prepended to the source sentence.

In contrast to the reinforcement learning approaches, both supervised baselines optimize for human-annotated edit suggestion quality directly but lack an appropriateness-specific training signal.

## 4 Data

This section briefly summarizes the datasets used in the studies and experiments below to model (1) inappropriateness and (2) human edits.

### 4.1 Inappropriateness data

The Appropriateness Corpus (Ziegenbein et al., 2023) comprises 2,191 arguments drawn from existing argument quality corpora, spanning three genres: debate portals, question answering forums, and reviews; manually annotated as appropriate (1,009 arguments) or inappropriate (1,182) with multi-label inappropriateness labels from a 14-dimensional taxonomy (see below). Each argument is annotated together with the issue it responds to. The annotators first judged whether they considered an argument inappropriate and, only if given, assigned further reasons from the taxonomy for that judgment. The corpus is the primary resource for training and evaluating the inappropriateness rewriting and editing approaches outlined in Section 3, and serves as the evaluation benchmark in this paper. To the best of our knowledge, it is also the only corpus annotated for inappropriateness that includes subdimensions, which is why all analyses in this paper are grounded in it. Ziegenbein et al. (2024) extended the corpus with 49,417 soft-labeled arguments from the Internet Argument Corpus v2 (Abbott et al., 2016) and from the GAQCorpus (Gretz et al., 2020) for training.

The taxonomy proposed by Ziegenbein et al. (2023) treats appropriateness as a minimal quality property that any argument must satisfy to be considered valuable in a debate, modeling it through the absence of flaws grounded in argument quality assessment and rhetoric. 14 dimensions are organized hierarchically under four core dimensions:

- *Toxic Emotions*, where emotional appeals are deceptive or too intense to allow critical evaluation;
- *Missing Commitment*, where an argument fails to engage seriously with the issue or to show openness to opposing views;
- *Missing Intelligibility*, where the meaning of an argument is unclear, irrelevant to the issue, or the reasoning difficult to follow; and
- *Other Reasons*, which captures orthographic issues and other cases that do not fit the previous three.

Each core dimension is further decomposed into subdimensions, nine in total, that we leave out here for lack of relevance. Our analysis focuses on the four core dimensions, as the edit intent labels collected in the moderator-style user study (Section 5) map directly onto them, making this the natural level of granularity for comparing revision behavior across approaches.

### 4.2 Human edit data

IteraTeR (Du et al., 2022) comprises 172,692 human edits from Wikipedia, ArXiv, and Wikinews, collected to study how humans revise text across domains. Ziegenbein et al. (2026) use the corpus to characterize natural human editing behavior for edit-based revision. We follow this practice to calibrate the thresholds and train the decoder language model underlying the conformity criteria Con_*a*_ and Con_*e*_ (see Section 6). We emphasize that the corpus is used exclusively for the structural signature of human editing. The pattern conformity model sequences of edit operations (keep, keep-in-edit, replace, delete, add), that is, how large edited spans are and how operations are composed and distributed. In contrast, the model deliberately abstracts away from *what* is being changed. The assumption is therefore not that Wikipedia or ArXiv editors revise for inappropriateness, but that human span-level editing shares a common structural grammar across revision goals. Whether an edit suggestion actually addresses inappropriateness is assessed separately by App_*e*_, which is trained on the moderator judgments collected in our user study (Section 5.5).

## 5 Moderator-style user study

We now report on a user study that we conducted for two purposes: first, to evaluate which edit suggestions from the different rewriting approach variants of Ziegenbein et al. (2024) human moderators find most appropriate for a given argument and edit intent; and second, to obtain annotator-assigned intent labels for individual edit suggestions, which serve as ground truth for creating and evaluating the edit-level appropriateness metric App_*e*_ (Section 5.5). No vulnerable populations were involved and no sensitive personal data was collected. All participants provided informed consent prior to participation.

### 5.1 Experimental setup

Five native English speakers (three female, two male) were recruited via Upwork.com and compensated at $15 per hour. Each participant was presented with all 200 inappropriate arguments from the Appropriateness Corpus, together with the discussion topic and the reason the argument was flagged as inappropriate. For each argument, edit suggestions from the rewriting approaches of Ziegenbein et al. (2024) were available, extracted via latexdiff as described in Section 3.1.

Participants interacted with a custom web-based annotation tool. The interface displayed the original argument on the left and the grouped edit suggestions on the right; edit operations affecting the same source span were grouped across all five approaches, enabling side-by-side comparison. Edit spans were color-coded by operation type: blue for replacements, red for deletions, and green for additions. Participants could click an edit suggestion to highlight its corresponding span in the argument text, or click an underlined span in the text to highlight the corresponding edit suggestion. The suggestions were labeled with anonymized approach names, so approach identity remained hidden from participants, and the competing suggestions within each group were presented in randomized order. Before starting, participants were presented with written guidelines describing the moderator role, the color coding, and the available actions (accept, dismiss, undo); they were instructed to improve each argument as much as possible while preserving its original gist. A screenshot of the annotation interface and the full annotation guidelines are provided in Supplementary material.

For each argument, edit suggestions were organized into *groups*: all edit suggestions from the five rewriting approaches targeting the same source span were collected into a single group. Groups were presented in order of their appearance in the argument but could be processed in any order, while the order of arguments was randomized across participants to reduce position and fatigue effects. For each group, participants repeated the following steps until all groups had been handled:

1. They selected exactly one edit suggestion from a group to apply, or dismissed the group entirely.
2. They assigned one or more *edit intents* from a predefined list: *Toxic Emotions, Missing Commitment, Missing Intelligibility*, or *Other*.
3. They confirmed their selected edit suggestion, which was then applied to the argument.

Once all groups had been processed, participants judged whether the resulting argument was fully appropriate (*yes*/*no*); if not, they indicated which parts remained problematic. Participants could undo any accepted or dismissed edit suggestion at any time, and annotations were saved incrementally to allow pausing and resuming across sessions.

### 5.2 Subjectivity of the editing task

Unlike categorical annotation tasks, editing is a sequential decision process: each accepted or dismissed edit changes the state of the argument and constrains subsequent choices. Accordingly, we do not interpret Krippendorff's α as a measure of annotation quality in the given setting, but rather as a tool for characterizing the inherent subjectivity of the task. Following Stab and Gurevych (2017) and Stahl et al. (2024), α is computed per token. At the macro level (edited vs. not-edited, regardless of approach), overall α across all five annotators is 0.426 (with 95% confidence interval [0.388, 0.462], bootstrap over arguments), indicating moderate agreement on *where* edit suggestions are needed. At the fine-grained level (which specific formulation was selected), α drops to 0.296 (with [0.263, 0.329]), reflecting that, while annotators converge on problematic spans, the choice among edit suggestions is more subjective. These values are in the range typically observed for subjective annotation tasks in computational argumentation. Wachsmuth et al. (2017) report α = 0.36 and Ziegenbein et al. (2023) α = 0.45 for inappropriateness judgments, and we interpret these values as evidence of a shared view on inappropriateness together with genuine disagreement at the margins, rather than of a universal standard. Crucially, however, the lower fine-grained agreement does not imply that individual annotation choices are arbitrary: in 784 out of 990 cases [79.2%, 95% CI [66.6, 91.2]], each annotator's personal selection of edit suggestions was sufficient to produce a fully appropriate argument, demonstrating that multiple editing strategies are viable paths to the same goal. On average, annotators applied 3.67 edit suggestions per argument, with an average edit span length of 16.7 words, suggesting that mitigating inappropriateness in deliberative arguments typically requires multiple targeted local changes rather than a single global correction.

### 5.3 Edit suggestion: structure and selection

Across all annotators and arguments, a total of 3,665 edit suggestions were selected and applied. Table 2 shows the edit suggestion structure and selection frequencies per rewriting approach. Each edit suggestion is decomposed into five mutually exclusive token categories: tokens outside the edit span that are kept unchanged (*KoE*), tokens inside the span that are kept (*KiE*), and tokens that are replaced (*Rep*), deleted (*Del*), or added (*Add*)—all five summing up to 100%. We observe that the more appropriateness-focused approaches (+PPO_*app*_, +PPO_*app*>*sim*_) produce substantially longer and more addition-heavy edit suggestions, while approaches that weight semantic similarity higher (+PPO_*app*<*sim*_, *Alpaca*) tend toward shorter, more conservative changes with a higher proportion of kept tokens. In absolute terms, *Alpaca* was selected most often (1,032 times). However, when normalizing by the number of candidate edits each approach offered, +PPO_*app*_ displays the highest selection rate (29.7%).

**Table 2:** User study results on rewriting approaches.

Approach	(a) Edit suggestion structure						(b) Selection frequencies		
	# Words ±σ	KoE	KiE	Rep	Del	Add	# Edits	Rate	App
Alpaca	8.4 ±13.0	82.8%	1.5%	6.3%	1.2%	8.2%	**1,032**	21.8%	70.6%
+ PPO_*app*_	**31.6** ±**34.2**	55.8%	**2.3%**	**16.8%**	2.9%	**22.2%**	821	**29.7%**	78.9%
+ PPO_*app*>*sim*_	23.5 ±31.3	66.7%	2.0%	14.7%	**3.4%**	13.1%	762	25.2%	**81.1%**
+ PPO_*app* = *sim*_	11.2 ±14.8	78.7%	1.4%	6.8%	2.9%	10.2%	576	14.7%	69.1%
+ PPO_*app*<*sim*_	4.6 ±8.5	**89.6%**	0.8%	4.0%	**3.4%**	4.5%	472	13.7%	67.4%

(a) Edit suggestion structure of the base LLM Alpaca and the PPO variants with different reward weightings: Mean edit span length in *# words*; proportion of tokens kept outside the edit span (*KoE*), kept inside (*KiE*), replaced (*Rep*), deleted (*Del*), or added (*Add*) respectively; (b) Selection frequencies: *# edits* selected by annotators, selection *rate* (selected/candidate edits), and proportion of selected edits in arguments judged fully appropriate (*App*). Bold values indicate the best value in each column.

These selection patterns, however, stand in tension with appropriateness: *Alpaca* is selected most often in absolute terms, yet achieves a much lower success rate in making an argument appropriate (70.6%). In contrast, +PPO_*app*>*sim*_ is selected less frequently but yields the highest success rate (81.1%). This suggests that what annotators choose and what actually resolves inappropriateness are not fully aligned.

To examine whether this disconnect is driven by the structural properties of edit suggestions themselves, we train two complementary logistic regression models, both using the five-category token breakdown as predictors: Since the five proportions in Table 2a sum up to one, we use *KoE* as the reference category and enter the remaining four (*KiE, Rep, Del, Add*) as predictors after *z*-scoring. *f*_*sel*_ predicts whether an edit suggestion is selected by an annotator (*n* = 17,906); *f*_*app*_ predicts whether a selected edit suggestion was part of an edit suggestion set that led to a fully appropriate argument (*N* = 3, 663). Both models include cluster-robust standard errors with 200 clusters (one per argument) to account for within-argument correlation across annotators and edit positions. In addition, both models constitute a secondary, exploratory analysis motivated by the descriptive tension reported above; no hypotheses about individual coefficients were specified in advance. We therefore report *p*-values as descriptive measures of association strength alongside the 95% confidence intervals, without correction for multiple comparisons. Because the data is cross-classified in arguments and annotators, we re-estimate both models with annotator-fixed effects, with two-way cluster-robust standard errors (argument × annotator), and as a crossed random-intercept model. For selection, we additionally estimate a conditional logit that conditions on the choice within each group of edit suggestions competing for the same span, which absorbs group-, argument-, and annotator-level confounding by design. All three cross-classified specifications preserve the coefficient signs reported below, and annotator-fixed effects also preserve their significance; under two-way clustering, which rests on only five annotator clusters and is correspondingly imprecise, the smaller selection-side effects lose significance. The conditional logit agrees on all coefficients except for *Add*, whose weak negative association with selection does not persist within groups, as discussed below. Full results are given in Supplementary material.

Figure 3 shows the coefficient estimates and 95% confidence intervals; throughout, β denotes the standardized logistic regression coefficient and *p* the two-sided *p*-value from the cluster-robust Wald test. We find that *Rep* is positively associated with both selection and appropriateness: edit suggestions that substitute existing text rather than merely preserving it are preferred by annotators *and* more likely to produce appropriate arguments. *KiE* is positively associated with selection (β>0, *p* = 0.027; β>0, *p* < 0.001 in the within-group model) but shows no significant association with appropriateness, indicating that edit suggestions which preserve substantial material within the span are judged as good choices. Yet, this preference does not independently predict argument-level success once other predictors are controlled for. The most informative pattern concerns *Add*: *Add* is negatively associated with selection (β < 0, *p* = 0.084) but positively associated with appropriateness (β>0, *p* = 0.011): addition-heavy edit suggestions gain no advantage at selection time, whereas revisions containing selected edits with larger addition proportions were more likely to receive a successful final appropriateness judgment. The within-group model qualifies this further: the weak negative association at selection time does not persist when annotators choose among competing suggestions for the same span (β≈0, *p* = 0.63), indicating that it reflects the dismissal of whole groups rather than a preference against additions among alternatives. These results are consistent with a tension between annotator preference and appropriateness, as observed in Table 2: edit suggestions with a higher proportion of added tokens were positively associated with argument-level appropriateness, yet annotators did not favor them at selection time.

**Figure 3:**
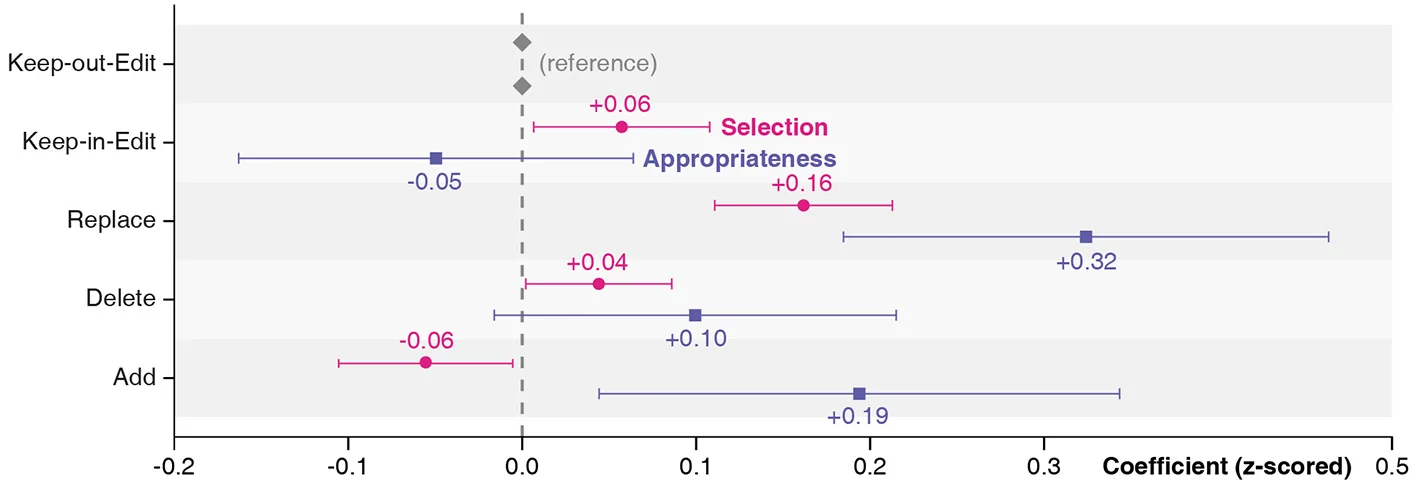
Logistic regression results: Forest plot of logistic regression coefficients for *f*_*sel*_ (edit suggestion selection) and *f*_*app*_ (edit suggestion appropriateness): predictors are *z*-scored token fractions with KoE as reference; error bars show cluster-robust 95% confidence intervals.

### 5.4 Edit suggestions: categories and intents

Table 3 characterizes the four annotator-assigned intents in terms of the selected proportions of the five edit suggestion categories. Edit suggestions targeting *toxic emotions* (34.3 words on average) and *missing commitment* (28.6 words) are substantially longer than those targeting *missing intelligibility* (13.0 words). This length difference is accompanied by a structural shift: Edit suggestions addressing toxic emotions (*Add* 23.0%) and missing commitment (*Add* 20.8%) devote substantially more argument tokens to additions than those addressing missing intelligibility (*Add* 10.1%). Replacement is the dominant active operation across the three main intents—toxic emotions (*Rep* 20.3%), missing commitment (*Rep* 16.8%), and missing intelligibility (*Rep* 8.4%)—while *other reasons* covers a heterogeneous set of cases and is excluded from this breakdown; but the deletion fraction further distinguishes toxic emotions from missing commitment: toxic emotions edit suggestions involve more deletion (*Del* 4.1% vs. 3.2%) and less within-span preservation, reflecting the need to excise emotionally charged language rather than augment commitment. Edit suggestions targeting missing intelligibility are structurally conservative, with most argument tokens untouched (*KoE* 78.5%) and only modest replacement (*Rep* 8.4%), consistent with targeted surface clarifications rather than structural rewrites.

**Table 3:** User study results on intent categories.

	(a) Presence		(b) Token proportions (%)					(c) Kendall's τ			
Intent	# Edits	# Words ±σ	KoE	KiE	Rep	Del	Add	TE	MC	MI	OR
Toxic Emotions (TE)	761	**34.3**±**34.3**	**49.8**	**2.7**	**20.3**	**4.1**	**23.0**	–	**0.125**	–0.274	**–0.087**
Missing Commitment (MC)	1,313	28.6 ± 31.9	56.5	2.6	16.8	3.2	20.8	**0.125**	–	–0.606	–0.123
Missing Intelligibility (MI)	**2,425**	13.0 ± 23.6	**78.5**	1.4	8.4	1.6	10.1	–0.274	–0.606	–	–0.188
Other Reasons (OR)	0,102	4.3 ± 7.9	93.9	0.3	1.0	2.2	2.6	–0.087	–0.123	**–0.188**	–

(a) Presence of the four intents in terms of *#edits* (multi-intent edits counted once per intent) and mean edit span length in *# words*; (b) proportion of argument tokens per operation type (*KoE/KiE/Rep/Del/Add*); (c) Kendall's τ rank correlation between row and column intent indicators across edits (diagonal:—). The best value in each column is marked bold.

,Among all edit suggestions, 77.7% carry a single intent, while 22.3% are assigned two or three intents simultaneously. Table 3c reports the Kendall's τ correlation between intent label pairs. *Toxic emotions* and *missing commitment* show a slight positive co-occurrence (τ = 0.125), while *missing intelligibility* correlates negatively with both *toxic emotions* (τ = −0.274) and *missing commitment* (τ = −0.606). This pattern reflects the different scopes of the intents: missing intelligibility edit suggestions are often narrow surface corrections, while those addressing toxic emotions and missing commitment tend to involve broader structural changes that co-address multiple dimensions.

Figure 4 extends this picture by comparing token proportions across single-intent, double-intent, and triple-intent edit suggestions. Multi-intent edit suggestions are consistently longer and more addition-heavy than single-intent ones: For *missing commitment*, mean edit suggestion length nearly doubles from single-intent (17.8 words) to multi-intent (34.6 words), and the addition fraction increases from 17.9% to 33.3%. The high keep fraction in single-intent *missing intelligibility* edit suggestions (*KoE* 87.9%) shrinks to 46.9% in multi-intent *missing intelligibility* edit suggestions, indicating that multi-intent edit suggestions require substantially more structural intervention. These patterns suggest that multi-intent edit suggestions address problems that resist targeted single-operation fixes, and may partly explain their lower selection rate—annotators appear to prefer the simpler, more bounded single-intent edit suggestions.

**Figure 4:**
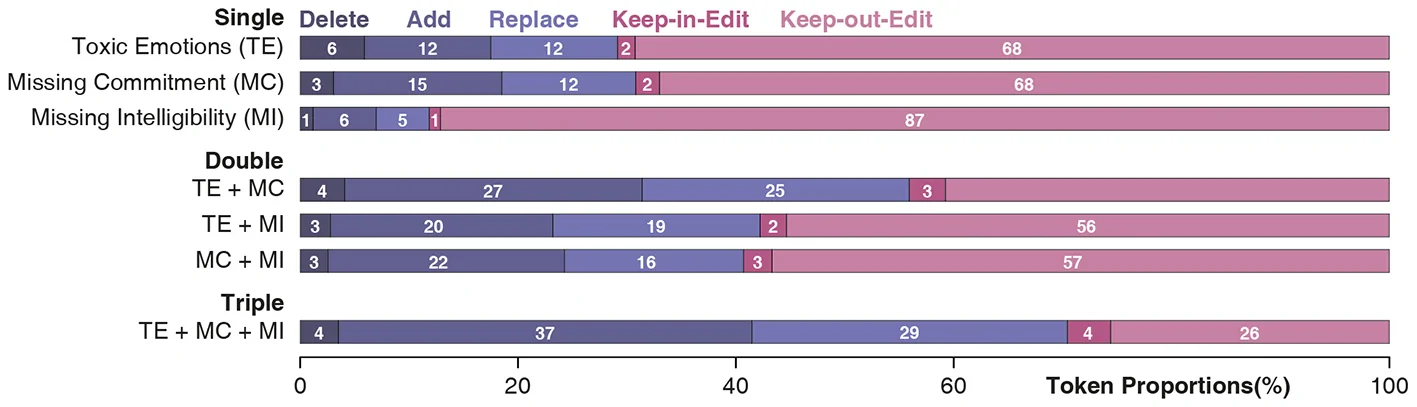
User study results on intent combinations: Token proportion breakdown per group (stacked bars; left to right: *Del, Add, Rep, KiE, KoE*). Groups are labeled by number of assigned intents (*single, double, triple*); single-intent suggestions show higher keep fractions, while multi-intent suggestions show markedly more addition and replacement, reflecting broader structural interventions.

### 5.5 Edit intent prediction

We investigate whether edit intent labels can be predicted automatically from an edit suggestion and its corresponding argument, framing this as a multi-label classification task over the four intent categories. We split the 3,665 annotated arguments into a training set (80%), validation set (10%), and a test set (10%). This yields 2,906 training, 405 validation, and 354 test edit suggestions, while ensuring that all edit suggestions from the same argument appear in the same split.

We compare the following eight approaches (full implementation details are given in Supplementary material):

- *Random*. A non-informative multi-label baseline that predicts each of the four intent labels independently with probability *p* = 0.5, regardless of the edit suggestion or argument.
- *Majority*. A frequency baseline that always predicts *Missing Intelligibility* (the most frequent label at 66.9% of training edit suggestions), regardless of the edit suggestion or argument.
- *Delta Replace* applies the appropriateness classifier of Ziegenbein et al. (2023) to the revised argument *a*^repl^ = apply(*a*, {*e*}) and computes $\Delta_{d}^{\text{repl}}=P_{d}\left(a\right)-P_{d}\left(a^{\text{repl}}\right)$, where *P*_*d*_(·) denotes the predicted probability for dimension *d*; all intents whose corresponding dimension shows a positive delta are predicted.
- *Delta Mask*. As above, but this baseline replaces the source span with [MASK], i.e., *a*^mask^ = apply(*a*, {(*s*, [MASK])}), computing $\Delta_{d}^{\text{mask}}=P_{d}\left(a\right)-P_{d}\left(a^{\text{mask}}\right)$; isolates the contribution of the removed span rather than the full edit suggestion.
- *Gemini 2.5* (Comanici et al., 2025) prompted zero-shot to predict the edit intent from the source and target spans, with (+* Argument*) and without the full argument as context.
- *ModernBERT* (Warner et al., 2025) fine-tuned on the edit spans of the 2,906 training edit suggestions for multi-label intent classification, with (+* Argument*) and without the argument as context.

Table 4 reports per-category F_1_-score, aggregate macro and micro F_1_-score, and Jaccard similarity between predicted and gold label sets (Jaccard measures set overlap and jointly penalizes missed and spurious labels). All LLM predictions are obtained with greedy decoding (no temperature sampling), so no sampling variation at generation time enters the comparison. To quantify the remaining test-set sampling variability, we compute 95% confidence intervals for every value by non-parametric bootstrap over the 354 test edit suggestions (2,000 resamples), and compare systems using paired resamples; the full interval and comparison tables are given in Supplementary material. We see that *delta replace* and *delta mask* remain close to the *random* baseline in macro *F*_1_ (0.439/0.467 vs. 0.454) and fall clearly below it in micro *F*_1_ (0.304/0.352 vs. 0.388) and Jaccard similarity (0.181/0.209 vs. 0.267), indicating that argument-level probability shifts are too diffuse a signal for reliable multi-label intent prediction at the edit suggestion level. Zero-shot *Gemini 2.5* outperforms the non-learning baselines on all three aggregate metrics (macro *F*_1_: 0.554; micro *F*_1_: 0.639; Jaccard: 0.568; all paired bootstrap tests *p* ≤ 0.027). It remains below fine-tuned *ModernBERT* in macro *F*_1_ (–0.061 without and –0.120 with argument context, both *p* < 0.001), whereas the differences in micro *F*_1_ and Jaccard similarity are not significant. *Gemini 2.5* in fact attains the highest values of any approach on these two metrics, indicating that it is accurate on the frequent intents while *ModernBERT* is more balanced across categories. Notably, adding argument context does not help and slightly degrades performance (macro *F*_1_: 0.532; micro *F*_1_: 0.602, *p* = 0.007; Jaccard: 0.524, *p* = 0.009). Fine-tuned *ModernBERT* with argument context is the best approach in macro *F*_1_ (0.652), the metric that weights all four intents equally, and improves over its span-only counterpart (+0.036, *p* = 0.045). *F*_1_ on *other reasons* is low for all other approaches, as expected given its role as a catch-all for cases that do not fit any of the three main intents, and consistent with the low *F*_1_ even in the argument-level classifier of Ziegenbein et al. (2023); fine-tuning with argument context is the only setting that handles this category noticeably better (0.601). *ModernBERT* with argument context, as the best-performing classifier, is subsequently used to instantiate App_*e*_ (Section 6). When App_*e*_ is applied to score the edit suggestions, we prevent information leakage through argument-level 5-fold cross-validation: the 200 arguments are partitioned into five folds, keeping all edit suggestions of an argument in the same fold, and five classifiers are trained, each on four of the folds. Every argument's edit suggestions are then scored only by the classifier that held that argument out.

**Table 4:** Edit intent prediction results: F_1_-score per intent label as well as micro F_1_, macro F_1_, and Jaccard similarity for all eight evaluated approaches.

Method	Toxic emotions	Missing commitment	Missing intelligibility	Other	Macro *F*_1_	Micro *F*_1_	Jaccard
Random	0.469	0.491	0.499	0.355	0.454	0.388	0.267
Majority	0.423	0.389	0.377	0.494	0.421	0.535	0.524
Delta replace	0.487	0.386	0.492	0.392	0.439	0.304	0.181
Delta mask	0.506	0.450	0.543	0.369	0.467	0.352	0.209
Gemini 2.5	**0.723**	0.560	0.446	0.489	0.554	**0.639**	**0.568**
+ Argument	0.696	0.532	0.413	0.487	0.532	0.602	0.524
ModernBERT	0.685	0.645	0.641	0.494	0.616	0.616	0.545
+ Argument	0.706	**0.658**	**0.643**	**0.601**	**0.652**	0.621	0.544

The best value in each column is marked bold.

## 6 Metric implementations

The abstract quality criteria defined in Section 2.1 are operationalized through concrete metric implementations introduced here. We distinguish two groups corresponding to the two levels of the framework: argument-level quality criteria assess the revised argument as a whole; edit suggestion-level quality criteria assess individual edits. Table 5 provides an overview of all implementations; the following subsections describe each in detail.

**Table 5:** Metric implementations: Base models, formulas, training and calibration data, and source papers for all four argument-level and the four edit suggestion-level quality criteria.

Criterion	Base model	Formula	Data	Source
Sim_*a*_	BERTScore	$F_{1}\left(a,a^{\prime }\right)$	–	Ziegenbein et al., 2024
Flu_*a*_	GPT-2	PPL(*a*′)	–	Ziegenbein et al., 2024
Con_*a*_	Decoder LM	PPL(diff(*a, a*′))	IteraTeR	Our approach
App_*a*_	DeBERTa-v3	𝕀 ∧*P*(App_*a*_) ≤ 0.5]	App. Corpus	Ziegenbein et al., 2023
Sim_*e*_	EmbeddingGemma	$\cos\left(a_{i},a_{i}^{\prime }\right)>\tau$	IteraTeR (calib.)	Ziegenbein et al., 2026
Flu_*e*_	ModernBERT	$P\left(\text{flu}|a_{i},a_{i}^{\prime }\right)\geq 0.5$	IteraTeR + aug.	Ziegenbein et al., 2026
Con_*e*_	Decoder LM	$\text{PPL}\left(\text{diff}\left(a_{i},a_{i}^{\prime }\right)\right)<\theta$	IteraTeR	Ziegenbein et al., 2026
App_*e*_	ModernBERT	∃*d*∈*D*:*P*(*d*∣*e, a*)>0.5	App. Corpus	Our approach

Thresholds τ and θ for Sim_*e*_ and Con_*e*_ are calibrated at the 99th percentile of human edits from IteraTeR (Du et al., 2022); *a*_*i*_ denotes the sentence of *a* containing the span affected by edit suggestion *e*; *D* = {TE, MC, MI} denotes the three main inappropriateness dimensions.

### 6.1 Argument-level metric implementations

The four argument-level criteria of Section 2.1.2 are implemented as follows.

- App_*a*_ is implemented using the multi-label inappropriateness classifier of Ziegenbein et al. (2023): a DeBERTa-v3 (He et al., 2021) model trained on the Appropriateness Corpus to predict inappropriateness scores across all 14 dimensions of the taxonomy. The criterion measures the percentage of arguments for which the classifier's prediction flips from inappropriate to appropriate after revision, i.e., $P\left({\text{App}}_{a^{\prime }}\right)>0.5$ while *P*(App_*a*_) ≤ 0.5.
- Sim_*a*_ is implemented using BERTScore (Zhang et al., 2020), which captures semantic overlap through contextual embeddings rather than surface-level lexical matching. The criterion is computed as the rescaled $F_{1}\left(a,a^{\prime }\right)$ between the original argument *a* and the revision *a*′.
- Flu_*a*_ is implemented using GPT-2 (Radford et al., 2019) as a reference-free measure of surface fluency. The criterion is computed as PPL(*a*′), with lower values indicating more fluent text.
- Con_*a*_ extends the edit-level conformity approach of Ziegenbein et al. (2026) to the document level, using a decoder-only transformer language model trained on token-level edit sequences expressed as keep, keep-in-edit, replace, delete, and add operations from IteraTeR (Du et al., 2022). The token-level edit sequence diff(*a, a*′), computed across all edits in *E*, is scored by this model; the value is PPL(diff(*a, a*′)), with lower values indicating greater conformity to human editing patterns.

### 6.2 Edit-level metric implementations

The four edit-level criteria of Section 2.1.3 are instantiated as binary classifiers; HL_App_ is their conjunction, as given in Equation 5 tence of *a* containing the span of *e* and $a_{i}^{\prime }$ the corresponding revised sentence:


$$\begin{array}{l} {\text{HL}}_{\text{App}}\left(e,a\right):=\text{𝕀}\left({\text{Sim}}_{e}\left(e,a\right)\wedge {\text{Flu}}_{e}\left(e,a\right)\wedge {\text{Con}}_{e}\left(e,a\right)\wedge {\text{App}}_{e}\left(e,a\right)\right) \end{array}$$ (5)

The first three classifiers operate on the sentence pair $\left(a_{i},a_{i}^{\prime }\right)$ rather than the full argument, as similarity, fluency, and conformity signals are local to the edited sentence.

- Sim_*e*_ is implemented using Google's EmbeddingGemma-300M (Schechter Vera et al., 2025) to measure cosine similarity between *a*_*i*_ and $a_{i}^{\prime }$ (Ziegenbein et al., 2026). An edit suggestion passes the criterion if $\cos\left(a_{i},a_{i}^{\prime }\right)>\tau$, where τ is the 1st percentile of semantic similarities computed on human edits from the IteraTeR dataset (Du et al., 2022), so that roughly 99% of human edits pass.
- Flu_*e*_ is implemented as a ModernBERT (Warner et al., 2025) classifier trained to detect fluency degradation (Ziegenbein et al., 2026). The classifier takes as input the sentence pair $\left(a_{i},a_{i}^{\prime }\right)$ and outputs a probability $P\left(\text{flu}|a_{i},a_{i}^{\prime }\right)\in \left[0,1\right]$; the edit suggestion passes if this probability is at least 0.5.
- Con_*e*_ follows the approach of Ziegenbein et al. (2026), using a small decoder-only transformer language model trained on edit operation sequences from IteraTeR (Du et al., 2022). We recreate this model with a hyperparameter search that optimizes training configuration and decoder architecture, with the goal of increasing the sensitivity of the conformity signal. An edit suggestion passes the criterion if $\text{PPL}\left(\text{diff}\left(a_{i},a_{i}^{\prime }\right)\right)<\theta$, where θ is the 99th percentile of IteraTeR edit sequences, indicating conformity to human editing patterns.
- App_*e*_ is implemented using the ModernBERT intent classifier introduced in Section 5.5, fine-tuned to predict which inappropriateness dimensions an edit suggestion addresses. The edit suggestion passes the criterion if the classifier predicts at least one of the three main dimensions with probability above 0.5, i.e., ∃*d*∈*D*:*P*(*d*∣*e, a*)>0.5, where *D* = {TE, MC, MI}; *other reasons* is excluded as a catch-all category that does not correspond to a targeted appropriateness improvement.

## 7 Experiments and results

All approaches from Section 3 are applied to the test split of the Appropriateness Corpus from Section 4 and assessed using the quality criteria from Section 6. We first examine edit-level quality and human-likeness pass rates across all approaches, then validate argument-level metrics against edit volume, compare approaches on the full argument-level metric suite, and finally study the effect of iterative application. Accordingly, argument-level metrics are reported under two views:

- *Arg-Level (All)* includes all generated edit suggestions;
- *Arg-Level (*HL_App_*)* is restricted to edit suggestions passing the extended human-likeness filter, which augments the standard filter with an edit-level appropriateness criterion (Section 2.1).

### 7.1 Edit-level quality

Table 6 presents edit-level quality results for all approaches. Edit-level appropriateness is consistently high across all paradigms (App_*e*_: 0.87–0.99), indicating that generated edit suggestions reliably improve the appropriateness of the targeted argument span. However, the human-likeness pass rate, defined as the fraction of edit suggestions satisfying all four criteria simultaneously, varies dramatically (HL_App_: 0.023–0.524), revealing that edit-level appropriateness alone does not guarantee human-likeness.

**Table 6:** Edit-level evaluation results of all rewriting and editing approaches along with the prompting and supervised learning baselines.

Approach	Setup			Edit-level					
	Output	Params	Training	Sim_*e*_	Flu_*e*_	Con_*e*_	App_*e*_	HL_App_	#HL_App_
RL – Rewriting approaches									
PPO_*app*_	Rewrite	7B	App. Corpus	0.335	0.624	0.525	0.992	0.088	68
PPO_*app*>*sim*_	Rewrite	7B	App. Corpus	0.382	0.576	0.530	0.991	0.114	67
PPO_*app* = *sim*_	Rewrite	7B	App. Corpus	0.570	0.492	0.653	0.970	0.167	120
PPO_*app*<*sim*_	Rewrite	7B	App. Corpus	0.782	0.613	0.825	0.984	0.393	175
RL – Editing approaches									
GRPO_*app*_	Edits	8B	App. Corpus	0.662	0.489	0.327	0.950	0.098	139
GRPO_*con*_	Edits	8B	App. Corpus	0.729	0.446	0.662	0.933	0.191	420
GRPO_*flu*_	Edits	8B	App. Corpus	0.610	0.872	0.059	0.960	0.023	24
GRPO_*sim*_	Edits	8B	App. Corpus	0.951	0.366	0.666	0.943	0.245	684
GRPO_¬*con*_	Edits	8B	App. Corpus	0.926	0.838	0.338	0.913	0.241	321
GRPO_¬*flu*_	Edits	8B	App. Corpus	0.953	0.348	0.787	0.944	0.257	855
GRPO_¬*sim*_	Edits	8B	App. Corpus	0.700	0.774	0.436	0.921	0.229	339
GRPO_*full*_	Edits	8B	App. Corpus	0.915	0.757	0.600	0.900	0.390	689
Prompting baselines									
Alpaca	Rewrite	7B	–	0.647	0.594	0.692	0.984	0.295	201
Llama	Edits	8B	–	0.823	0.659	0.589	0.871	0.311	552
Gemini 2.5	Edits	–	–	0.795	0.656	0.731	0.946	0.421	598
GPT-5	Edits	–	–	0.935	0.675	0.832	0.921	0.524	1397
Supervised baselines									
DeLIteraTeR	Edits	1B	IteraTeR	0.845	0.475	0.754	0.958	0.273	218
CoEdIT	Rewrite	11B	IteraTeR	0.993	0.583	0.951	0.955	0.510	377

*output*, type of generated output; *params*, parameter count; *training*, task-specific training data used; *Sim*_*e*_, semantic similarity between source and target span; *Flu*_*e*_, local sentence fluency after the edit; *Con*_*e*_, pattern conformity to human edit distributions; *App*_*e*_, edit-level appropriateness improvement; *HL*_App_, fraction of edits passing all four criteria simultaneously; *#HL*_App_, absolute count of passing edits. Underline: best in group; bold: best overall.

For the *RL – Rewriting* approaches, the human-likeness pass rate increases monotonically with the PPO similarity weight α: *PPO*_*app*<*sim*_ achieves the highest pass rate in this group (HL_App_ = 0.393), driven by strong similarity and pattern conformity (Sim_*e*_ = 0.782, Con_*e*_ = 0.825), while *PPO*_*app*_ retains only 0.088 (HL_App_) of its edit suggestions, as unconstrained appropriateness optimization substantially erodes both. The *RL – Editing* paradigm reveals an even starker pattern: *GRPO*_*flu*_ achieves the highest local fluency of any approach (Flu_*e*_ = 0.872) but near-zero pattern conformity (Con_*e*_ = 0.059), yielding the lowest overall human-likeness pass rate (HL_App_ = 0.023), demonstrating that optimizing a single reward component can simultaneously collapse the others. *GRPO*_*full*_, which combines all reward components, achieves the highest human-likeness pass rate within the RL editing group (HL_App_ = 0.390; *n*_HL_ = 689), confirming that multi-component reward structures are beneficial for balanced edit quality. Among the *prompting* baselines, *GPT-5* achieves the highest human-likeness pass rate of any approach (HL_App_ = 0.524; *n*_HL_ = 1, 397), combining strong similarity, fluency, and pattern conformity (Sim_*e*_ = 0.935, Flu_*e*_ = 0.675, Con_*e*_ = 0.832). Within the *supervised* baselines, *CoEdIT* achieves the highest similarity and pattern conformity of any approach (Sim_*e*_ = 0.993, Con_*e*_ = 0.951), reflecting that its sequence-to-sequence training closely mirrors human span-level edit suggestion distributions, while *DeLIteraTeR* exhibits comparatively low local fluency (Flu_*e*_ = 0.475). Across paradigms, the reinforcement-learned rewriting approaches show lower local fluency than the baselines, suggesting that the rewrite–differencing pipeline, rather than the underlying model, is a primary contributor to fluency degradation in this paradigm.

Table 7 shows, using examples from different GRPO approaches of Ziegenbein et al. (2026), edit suggestions that pass all human-likeness criteria: these are typically short, locally conservative replacements that remove inappropriate phrasing while preserving the argument's structure and meaning. Table 8 complements this by illustrating how each criterion captures a distinct failure mode: edit suggestions with low pattern conformity span entire sentences in ways that deviate from typical human editing patterns; edit suggestions with low fluency produce sentences that no longer read naturally after application; and edit suggestions with low semantic similarity replace substantial content rather than making targeted corrections.

**Table 7:** Example edits from GRPO approaches of Ziegenbein et al. (2026), passing all HL_App_ criteria (*Con*_*e*_, *Flu*_*e*_, *Sim*_*e*_, *App*_*e*_): red = source span; blue = target span.

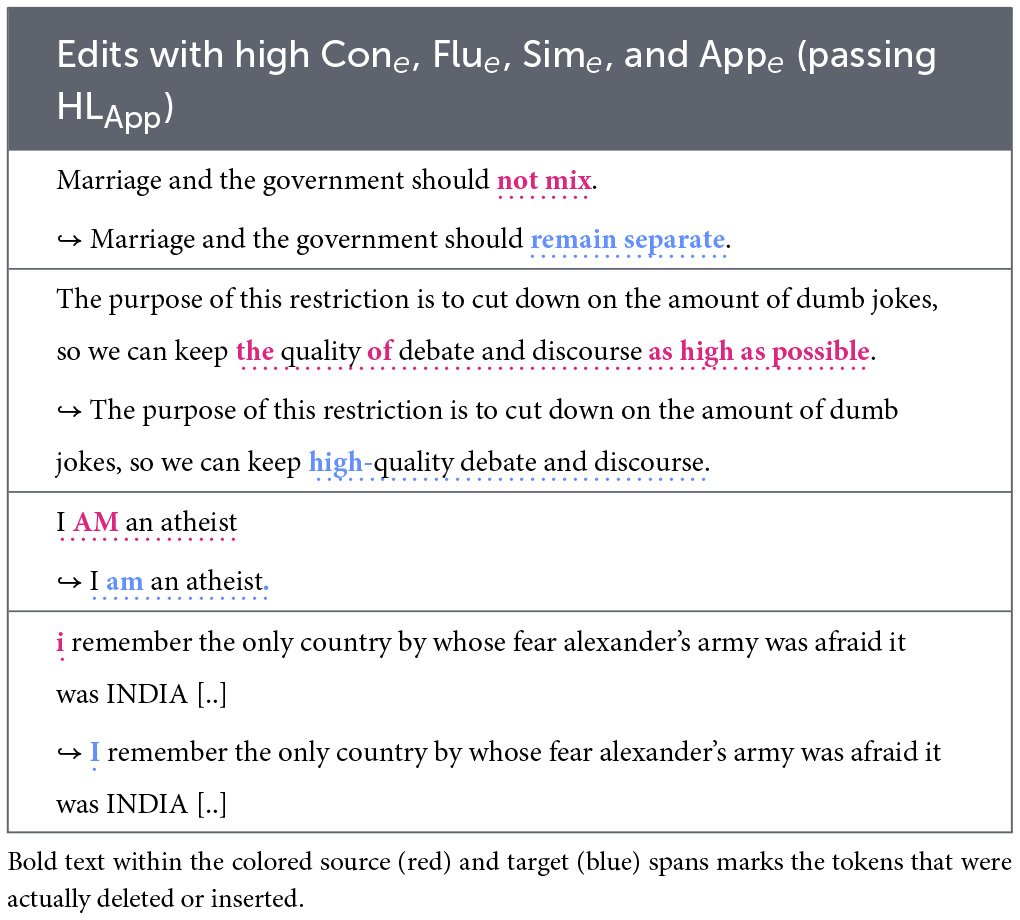

Bold text within the colored source (red) and target (blue) spans marks the tokens that were actually deleted or inserted.

**Table 8:** Example edits from GRPO approaches of Ziegenbein et al. (2026), illustrating failures in individual quality dimensions (*Con*_*e*_, *Flu*_*e*_, *Sim*_*e*_): red = source span; blue = target span.

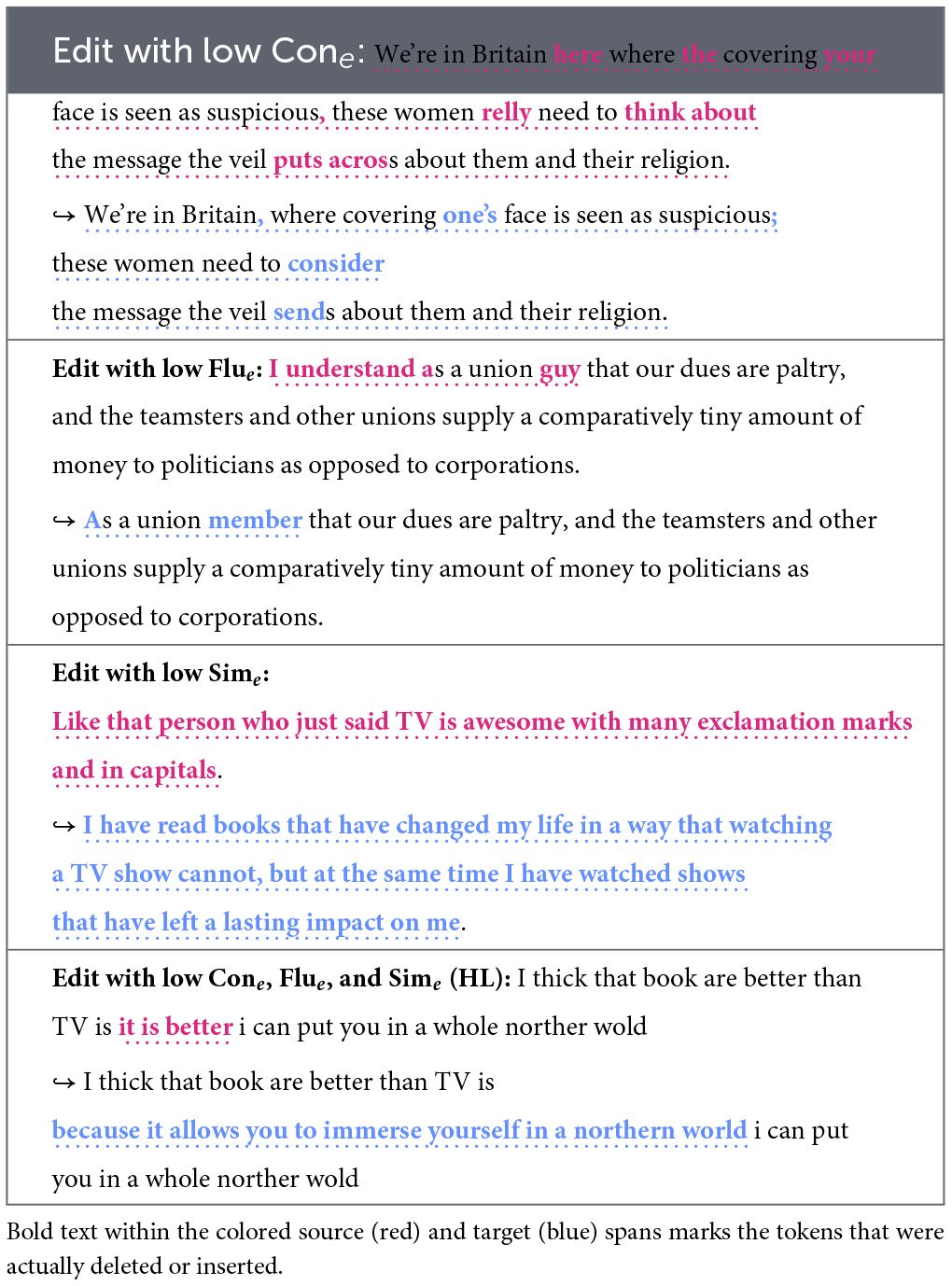

Bold text within the colored source (red) and target (blue) spans marks the tokens that were actually deleted or inserted.

### 7.2 Argument-level metric validation

Argument-level metrics aggregate over all edit suggestions of an approach, which raises the question of whether differences between approaches reflect edit quality or merely edit suggestion volume: an approach generating more edit suggestions has more opportunities to improve appropriateness, potentially inflating aggregate scores regardless of per-edit quality. We examine whether argument-level metrics are confounded with the number of quality-filtered edits per approach using Kendall's τ rank correlation (Table 9). While App_*a*_ also correlates positively with edit suggestion volume (τ = 0.611), which is expected since more edit suggestions plausibly improve appropriateness, Con_*a*_ shows a notable negative correlation with volume (τ = −0.386), indicating that Con_*a*_ penalizes approaches that produce more edit suggestions. Such approaches tend to achieve stronger appropriateness improvement, thereby biasing the aggregate score toward approaches that produce less appropriate revisions. To mitigate this confound, we introduce All_τ_, a three-metric geometric mean excluding Con_*a*_:


$${\text{All}}_{\tau }={\left({\text{Sim}}_{a}\times \frac{1}{{\text{Flu}}_{a}}\times {\text{App}}_{a}\right)}^{1/3}$$

This score removes the volume bias introduced by Con_*a*_ while retaining the multi-dimensional evaluation framework, and coincides with the aggregate metric proposed by Ziegenbein et al. (2026), whose manual evaluation demonstrates that it aligns with human preference. Both All (4-metric) and All_τ_ (3-metric) are reported in Table 10. We further discuss the implications of Con_*a*_ and All in Section 8.

**Table 9:** Kendall's τ pairwise rank correlations between argument-level metrics and edit volume (#HL_App_).

Dimension	Kendall's τ				
	#HL_App_	Sim_a_	Flu_a_	Con_a_	App_a_
#HL_App_		–0.634	**0.647**	–0.386	0.611
Semantic Similarity (Sim_*a*_)	–0.634		–0.569	**0.490**	–0.758
Fluency (Flu_*a*_)	**0.647**	–0.569		–0.634	0.518
Pattern Conformity (Con_*a*_)	–0.386	**0.490**	–0.634		-0.492
Appropriateness (App_*a*_)	**0.611**	–0.758	0.518	–0.492	

Bold: strongest positive τ per row; underline: strongest negative τ per row.

**Table 10:** Argument-level evaluation results of all approaches and baselines, reported for all generated edits (*Arg-Level (All)*) and for HL_App_-filtered edits (*Arg-Level (*HL_App_*)*).

Approach	Arg-level (all)						Arg-level (HL_App_)					
	Sim_a_	Flu_a_↓	Con_a_↓	App_a_	All	All_τ_	Sim_a_	Flu_a_↓	Con_a_↓	App_a_	All	All_τ_
RL – Rewriting approaches												
PPO_*app*_	0.191	18.3	1.764	0.720	0.255	0.196	0.961	83.2	1.280	0.262	0.221	0.145
PPO_*app*>*sim*_	0.298	24.7	1.639	0.547	0.252	0.188	0.959	82.8	1.312	0.271	0.221	0.146
PPO_*app* = *sim*_	0.436	27.3	1.626	0.551	0.271	0.206	0.950	80.6	1.228	0.284	0.228	0.150
PPO_*app*<*sim*_	0.829	45.6	1.429	0.289	0.246	0.174	0.963	72.3	1.273	0.271	0.231	0.153
RL – Editing approaches												
GRPO_*app*_	0.326	20.0	2.496	0.898	0.277	0.245	0.955	70.9	1.475	0.249	0.218	0.150
GRPO_*con*_	0.292	31.2	2.596	0.902	0.239	0.204	0.881	66.3	1.485	0.302	0.228	0.159
GRPO_*flu*_	0.465	22.7	2.352	0.787	0.288	0.253	0.989	85.8	1.368	0.253	0.215	0.143
GRPO_*sim*_	0.376	27.0	2.340	0.773	0.260	0.221	0.898	63.0	1.523	0.307	0.232	0.164
GRPO_¬*con*_	0.745	33.6	2.838	0.431	0.241	0.212	0.941	64.8	1.685	0.276	0.221	0.159
GRPO_¬*flu*_	0.406	36.2	2.481	0.760	0.242	0.204	0.869	64.6	1.636	0.324	0.227	0.163
GRPO_¬*sim*_	0.545	30.6	2.650	0.667	0.259	0.228	0.925	61.5	1.658	0.276	0.224	0.161
GRPO_*full*_	0.742	38.5	2.908	0.422	0.230	0.201	0.908	61.2	1.777	0.311	0.226	0.166
Prompting baselines												
Alpaca	0.619	35.2	1.557	0.329	0.247	0.180	0.952	84.3	1.258	0.262	0.220	0.144
Llama	0.707	48.7	2.752	0.404	0.215	0.180	0.922	62.2	1.650	0.280	0.224	0.161
Gemini 2.5	0.675	56.3	2.496	0.511	0.223	0.183	0.905	67.9	1.629	0.311	0.225	0.161
GPT-5	0.630	43.1	2.998	0.618	0.234	0.208	0.846	58.2	1.984	0.347	0.225	0.171
Supervised approaches												
DeLIteraTeR	0.716	54.1	1.486	0.236	0.214	0.146	0.971	79.0	1.181	0.258	0.228	0.147
CoEdIT	0.967	79.3	1.234	0.240	0.221	0.143	0.975	79.7	1.208	0.249	0.224	0.145

*All*, geometric mean ${\left({\text{Sim}}_{a}\times \left(1/{\text{Flu}}_{a}\right)\times \left(1/{\text{Con}}_{a}\right)\times {\text{App}}_{a}\right)}^{1/4}$; *All*_τ_, geometric mean excluding pattern conformity ${\left({\text{Sim}}_{a}\times \left(1/{\text{Flu}}_{a}\right)\times {\text{App}}_{a}\right)}^{1/3}$. Underline: best in group; bold: best overall.

### 7.3 Argument-level edit quality

Table 10 presents argument-level evaluation results across all approaches. Considering all edit suggestions (*Arg-Level (All)*), the training paradigm has a substantial effect on the trade-off between appropriateness improvement and content preservation. Within *RL – Rewriting, PPO*_*app*_ achieves the highest appropriateness (App_*a*_ = 0.720) but the lowest similarity (Sim_*a*_ = 0.191), reflecting unconstrained appropriateness optimization. *PPO*_*app*<*sim*_ inverts this pattern (Sim_*a*_ = 0.829, App_*a*_ = 0.289), while the balanced variant *PPO*_*app* = *sim*_ achieves the best aggregate score within this group (All = 0.271). The *RL – Editing* reward ablations reveal a complementary picture: *GRPO*_*app*_ and *GRPO*_*con*_ achieve the highest appropriateness (App_*a*_: 0.898 and 0.902, respectively), while *GRPO*_*flu*_ achieves the highest aggregate score across all approaches (All = 0.288; All_τ_ = 0.253). Across all reinforcement-learned approaches, argument-level pattern conformity is substantially higher for editing than for rewriting approaches (Con_*a*_: 2.3–2.9 vs. 1.4–1.8), indicating that edit suggestions generated directly as structured output deviate more from human editing patterns at the argument level than those derived ex-post via latexdiff. Among the *Prompting* baselines, *GPT-5* is competitive with the best reinforcement-learned rewriting variants (App_*a*_ = 0.618; All = 0.234) while also generating a large number of edit suggestions (*n* = 2, 664). The *supervised* baselines exhibit the opposite profile: *CoEdIT* achieves the highest (Sim_*a*_ = 0.967, Con_*a*_ = 1.234) of any approach, indicating that sequence-to-sequence edit suggestion generation closely mirrors human editing conventions, but at the cost of very low appropriateness (App_*a*_ = 0.240), confirming that a task-specific training signal is necessary for systematic appropriateness improvement.

The human-likeness filter (*Arg-Level (*HL_App_*)*) substantially changes the ranking. The fraction of edit suggestions passing all four quality criteria ranges from 0.023 (*GRPO*_*flu*_) to 0.524 (*GPT-5*), revealing that approaches solely focused on high appropriateness improvement frequently achieve it through edit suggestions that fail the human-likeness filter. For instance, *PPO*_*app*_ and *PPO*_*app*>*sim*_ have pass rates of only 0.088 and 0.114 (HL_App_), respectively. Under the human-likeness filter, semantic similarity converges to the 0.85–0.99 range across most approaches, as the filter selects edit suggestions that are locally conservative. *GPT-5* remains the top performer under the human-likeness filter (App_*a*_ = 0.347; All_τ_ = 0.171), indicating that large-scale instruction-following models can simultaneously satisfy human-likeness and appropriateness requirements. Among reinforcement-learned editing approaches, *GRPO*_*sim*_ achieves the highest quality-filtered aggregate score (All = 0.232). Supervised approaches, despite comparatively high pass rates (HL_App_: 0.273–0.510), yield the lowest quality-filtered appropriateness improvement (App_*a*_: 0.249–0.258), confirming that alignment with human editing conventions in general-domain training data does not transfer to appropriateness-specific improvement. We provide additional reports broken down by inappropriateness dimension in Supplementary material.

### 7.4 Iterative application

To study the effect of iteratively revising arguments with LLMs, we follow Ziegenbein et al. (2026) but adjust their experiment setup to our unified edit-operation framework. In particular, we reapply *GRPO*_*full*_ to its own output for 11 revision rounds on the test set, running until the proportion of arguments classified as appropriate converges. We note that this experiment is intended to illustrate what iterative application can achieve in principle rather than to prescribe an optimal number of rounds. Full per-round results are provided in Supplementary material.

Figure 5a shows that pattern conformity and semantic similarity remain nearly stable across revisions (ΔCon_*e*_ = +0.009, ΔSim_*e*_ = −0.044) and edit-level appropriateness improves marginally (ΔApp_*e*_ = +0.009), but local fluency and the human-likeness pass rate decrease considerably (ΔFlu_*e*_ = −0.289, ΔHL_App_ = −0.176): As the argument approaches appropriateness, remaining edits increasingly struggle to preserve local sentence coherence. Figure 5b shows that semantic similarity declines sharply (ΔSim_*a*_ = −0.243; under HL_App_: Δ = −0.361) as individually conservative edits accumulate into semantic drift, while argument-level appropriateness rises substantially (ΔApp_*a*_ = +0.237; under HL_App_: Δ = +0.299) and both argument-level fluency and pattern conformity show a consistent overall improvement (ΔFlu_*a*_ = −11.79, ΔCon_*a*_ = −1.78). The aggregate score improves substantially (ΔAll = +0.093; under HL_App_: Δ = +0.107), indicating that iterative application is effective overall, though at the cost of monotonically declining semantic similarity, as the cumulative effect of individually conservative edits is a gradual drift from the argument's original meaning. This is an inherent trade-off that will likely always remain.

**Figure 5:**
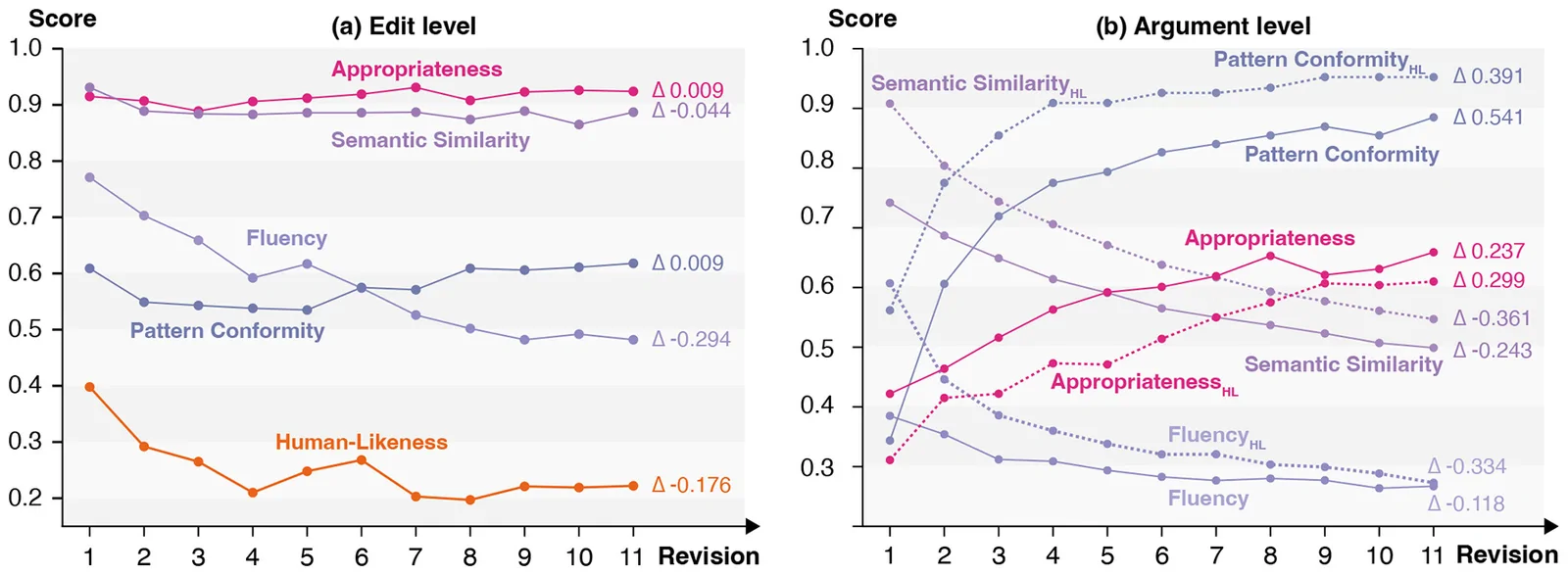
Iterative revision results across 11 rounds: **(a)** Edit-level results. **(b)** Argument-level results. Δ shows the difference between first and last rounds. Inspired by Ziegenbein et al. (2026).

## 8 Discussion

The proposed unified edit-operation framework enables a cross-paradigm analysis that neither prior work on argument rewriting nor prior work on span-level editing could offer individually. Analyzing both rewriting-derived and directly-generated edit suggestions within the same framework reveals that the two paradigms produce structurally different problems, and that the source of those problems lies in how edit suggestions are produced. Edit suggestions extracted *post-hoc* using a difftool carry a structural limitation invisible at the argument level: individual edit suggestions from the same rewrite are often interdependent, and selecting a subset breaks fluency, motivating the need for the span-level quality criteria introduced in this work. Directly-generated edit suggestions avoid this dependency but are less effective as standalone changes, a limitation that iterative application can address.

Our user study in Section 5 reveals a systematic divergence between what annotators prefer to select and what actually resolves inappropriateness. Annotators favor structurally conservative edit suggestions that preserve substantial material within the target span, yet the token-level regression analysis shows that edit suggestions with a higher proportion of added tokens are the strongest independent predictor of an argument reaching full appropriateness. The user study further reveals that inappropriateness dimensions impose qualitatively-different structural demands on edit suggestions. Edit suggestions addressing *toxic emotions* and *missing commitment* are markedly longer and devote a higher fraction of argument tokens to additions and replacements than edit suggestions targeting *missing intelligibility*, which is addressed by short, targeted surface corrections. Approach preferences in the user study align with these demands: appropriateness-optimized rewriting variants are preferred for toxic emotions and missing commitment, while an instruction-tuned baseline dominates for missing intelligibility, supporting the development of dimension-specific revision strategies.

Among the prompting-only approaches that we considered as baselines, model size appears to be associated with edit suggestion quality: larger proprietary models produce edit suggestions that are simultaneously more appropriate and more structurally human-like, achieving the highest human-likeness pass rates of any paradigm. This suggests that general-purpose instruction-following capability, when sufficiently scaled, transfers non-trivially to generating edit suggestions for inappropriate arguments, though it comes at the cost of computational resources that are inaccessible to task-specific training pipelines. The computational results, however, show that optimizing directly for human-likeness criteria, rather than for argument-level appropriateness alone, markedly improves the quality of suggestions generated directly The considered reinforcement learning approaches combining multiple reward components achieve human-likeness pass rates competitive with prompted models that are orders of magnitude larger, suggesting that the multi-component reward structure is consequential: rewarding appropriateness, fluency, and conformity simultaneously prevents the quality collapses observed under single-component ablations, where optimizing one criterion markedly degrades the others. In our experiments, applying a direct-generation model to its own output across multiple rounds, as would be the natural deployment setting, suggested that argument-level appropriateness improves monotonically toward levels achieved by the rewriting paradigm, while individual edit suggestion quality remains stable through early rounds. This convergence suggests that the two paradigms occupy different positions on a quality-volume trade-off: Rewriting produces fewer, larger edit suggestions with higher impact per edit suggestion, while direct generation produces more, smaller edit suggestions whose cumulative effect is comparable only after several rounds. The results of this work suggest the latter to be the preferable approach coinciding with the results of Ziegenbein et al. (2026), who compare the two paradigms directly in human evaluation. Given that the two paradigms are instantiated by approaches that differ in terms of their model, instruction tuning, optimization algorithm, output representation, and reward design, we interpret the paradigm differences as associative rather than strictly causal.

One finding that warrants separate discussion concerns the argument-level pattern conformity metric Con_*a*_. Although pattern conformity of argument structure is a conceptually-important quality dimension, the current operationalization yields results that are difficult to interpret in this setting: values increase alongside appropriateness improvement rather than decreasing, indicating that approaches producing more edit suggestions score higher on both dimensions simultaneously, and the metric appears sensitive to a domain mismatch between the data on which the underlying model was trained and the deliberative arguments used here. This operationalization should be treated as preliminary; future work needs to either retrain the pattern conformity model on in-domain deliberative text or replace it with a domain-appropriate alternative before drawing conclusions from Con_*a*_ values. We therefore do not recommend Con_*a*_ in its current form and instead recommend *All*_τ_ as the primary aggregate metric, as it captures the remaining quality dimensions without inheriting the domain mismatch that affects argument-level pattern conformity scoring.

## 9 Conclusion

Inappropriate arguments undermine the quality of public deliberation; automated revision support offers a scalable complement to human moderation. We have introduced a unified edit-operation framework that places rewriting-derived and directly-generated edit suggestions on a common footing. We have systematically demonstrated that this unification is necessary to reveal structural properties that are invisible to argument-level metrics alone: edit suggestion dependency, selection-effectiveness misalignment, and dimension-specific edit suggestion structure. The clearest result regarding these properties is that the two generation paradigms fail in different ways rather than to different degrees. Edit suggestions extracted from full rewrites are frequently interdependent, so that selecting a subset of them breaks fluency, whereas edit suggestions generated directly are self-contained but individually less effective and depend on iterative application to reach full appropriateness. Neither shortcoming is visible when revisions are scored only as whole arguments, which is what makes the edit-level view essential rather than merely complementary. Our moderator-style user study has revealed that human moderators can achieve full appropriateness in a majority of arguments when selecting from edit suggestions, while the computational experiment shows that optimizing directly for human-likeness criteria closes much of the quality gap between task-specific reinforcement-learned large language models (LLM) and prompted state-of-the-art LLMs. Multiple paradigms have now been analyzed under comparable conditions, yet their distinct strengths have not been fully combined; the most promising direction is a reinforcement-learned LLM approach that generates edit suggestions directly and optimizes jointly over fluency, conformity, and appropriateness. The evaluation framework introduced here is approach-agnostic and designed to remain useful as this line of research develops, providing a stable method for evaluation and supporting the design of dimension-specific, human-in-the-loop revision systems for AI-assisted deliberation.

Beyond its methodological contributions, this work speaks directly to the broader challenge of sustaining productive public deliberation in an era of large-scale online discourse. Inappropriate argumentation, which can manifest through emotional excess, lack of commitment, or reduced intelligibility, erodes the mutual understanding that deliberation requires, and automated revision support of the kind investigated here offers a means to address this at scale without replacing human judgment. The user study demonstrates that human moderators can effectively leverage AI-generated edit suggestions, positioning such approaches as collaborative tools that augment rather than supplant human oversight. The evaluation framework introduced here, built around empirically grounded, multi-dimensional quality criteria, provides a transparent and replicable basis for assessing the quality of AI-generated edit suggestions, which constitutes a prerequisite for trustworthy deployment in deliberative platforms. Taken together, these contributions advance the development of AI tools that support the quality of democratic discourse while preserving the interpretability and human agency that responsible governance of such approaches demands.

## Data Availability

The original contributions presented in the study are included in the article/Supplementary material, further inquiries can be directed to the corresponding author.

## Footnotes

1
https://ctan.org/pkg/latexdiff

2
https://ctan.org/pkg/latexdiff

## References

[B1] Abbott R. Ecker B. Anand P. Walker M. (2016). “Internet argument corpus 2.0: an SQL schema for dialogic social media and the corpora to go with it,” in Proceedings of the tenth international conference on language resources and evaluation (LREC'16), eds. N. Calzolari, K. Choukri, T. Declerck, S. Goggi, M. Grobelnik, B. Maegaard, et al. (Portoro: European Language Resources Association (ELRA)), 4445–4452.

[B2] Bhan M. Vittaut J.-N. Achache N. Legrand V. Blangero A. Chesneau N. . (2026). “Mitigating text toxicity with counterfactual generation,” in Explainable artificial intelligence (Cham: Springer Nature Switzerland), 135–157. doi: 10.1007/978-3-032-08330-2_7

[B3] Borkan D. Dixon L. Sorensen J. Thain N. Vasserman L. (2019). Nuanced metrics for measuring unintended bias with real data for text classification. CoRR, abs/1903.04561. doi: 10.48550/arXiv.1903.04561

[B4] Cabrio E. Villata S. (2018). “Five years of argument mining: a data-driven analysis,” in Proceedings of the 27th international joint conference on artificial intelligence (International Joint Conferences on Artificial Intelligence Organization, CA: IJCAI/AAAI Press), 5427–5433.

[B5] Chandrasekharan E. Samory M. Jhaver S. Charvat H. Bruckman A. Lampe C. . (2018). The internet's hidden rules: an empirical study of reddit norm violations at micro, meso, and macro scales. Proc. ACM Hum.-Comput. Interact. 2:32. doi: 10.1145/3274301

[B6] Christiano P. F. Leike J. Brown T. B. Martic M. Legg S. Amodei D. (2017). “Deep reinforcement learning from human preferences,” in Proceedings of the 31st international conference on neural information processing systems, NIPS'17 (Red Hook, NY: Curran Associates Inc.), 4302–4310.

[B7] Comanici G. Bieber E. Schaekermann M. Pasupat I. Sachdeva N. Dhillon I. . (2025). Gemini 2.5: pushing the frontier with advanced reasoning, multimodality, long context, and next generation agentic capabilities. Technical report, Google DeepMind. *arXiv:2507.06261*. Available online at: https://arxiv.org/abs/2507.06261

[B8] Dale D. Voronov A. Dementieva D. Logacheva V. Kozlova O. Semenov N. . (2021). “Text detoxification using large pre-trained neural models,” in Proceedings of the 2021 conference on empirical methods in natural language processing (Stroudsburg, PA: Association for Computational Linguistics), 7979–7996. doi: 10.18653/v1/2021.emnlp-main.629

[B9] Davidson T. Warmsley D. Macy M. Weber I. (2017). “Automated hate speech detection and the problem of offensive language,” in Proceedings of the 11th international AAAI conference on web and social media (ICWSM 2017), Vol. 11, (Palo Alto, CA: AAAI Press), 512–515. doi: 10.1609/icwsm.v11i1.14955

[B10] Du W. Raheja V. Kumar D. Kim Z. M. Lopez M. Kang D. (2022). “Understanding iterative revision from human-written text,” in Proceedings of the 60th annual meeting of the association for computational linguistics (volume 1: long papers) (Dublin: Association for Computational Linguistics), 3573–3590. doi: 10.18653/v1/2022.acl-long.250

[B11] Gorwa R. Binns R. Katzenbach C. (2020). Algorithmic content moderation: technical and political challenges in the automation of platform governance. Big Data Soc. 7:1–15. doi: 10.1177/2053951719897945

[B12] Grattafiori A. Dubey A. Jauhri A. . (2024). The llama 3 herd of models.

[B13] Gretz S. Friedman R. Cohen-Karlik E. Toledo A. Lahav D. Aharonov R. . (2020). “A large-scale dataset for argument quality ranking: construction and analysis,” in Proceedings of the AAAI conference on artificial intelligence, vol. 34 (Palo Alto, CA: AAAI Press), 7805–7813.

[B14] Habermas J. (1979). “What is universal pragmatics?,” in Communication and the Evolution of Society (Boston, MA: Beacon Press), 22, 1–68.

[B15] Habermas J. (1984). The *Theory of Communicative Action*, vol. 1. Boston, MA: Beacon Press, 286.

[B16] Habermas J. (1990). Moral Consciousness and Communicative Action. Cambridge, MA: MIT Press.

[B17] Habernal I. Gurevych I. (2016). “Which argument is more convincing? Analyzing and predicting convincingness of web arguments using bidirectional LSTM,” in Proceedings of the 54th annual meeting of the association for computational linguistics (volume 1: long papers) (Berlin: Association for Computational Linguistics), 1589–1599. doi: 10.18653/v1/P16-1150

[B18] Hallinan S. Liu A. Choi Y. Sap M. (2023). “Detoxifying text with MaRCo: controllable revision with experts and anti-experts,” in Proceedings of the 61st annual meeting of the association for computational linguistics (volume 2: short papers) (Toronto, ON: Association for Computational Linguistics), 228–242. doi: 10.18653/v1/2023.acl-short.21

[B19] He P. Gao J. Chen W. (2021). DeBERTaV3: improving DeBERTa using ELECTRA-style pre-training with gradient-disentangled embedding sharing. CoRR, abs/2111.09543. doi: 10.48550/arXiv.2111.09543

[B20] He X. Zannettou S. Shen Y. Zhang Y. (2024). “You only prompt once: on the capabilities of prompt learning on large language models to tackle toxic content,” in 2024 IEEE security and privacy workshops (SPW). San Francisco, CA, USA, May 23, 2024 (Piscataway, NJ: IEEE). arXiv:2308.05596.

[B21] Horta Ribeiro M. West R. Lewis R. Kairam S. (2025). Post guidance for online communities. Proc. ACM Hum.-Comput. Interact. 9, 1–26. doi: 10.1145/3711046

[B22] Hu E. J. Shen Y. Wallis P. Allen-Zhu Z. Li Y. Wang S. . (2022). “LoRA: low-rank adaptation of large language models,” in International conference on learning representations. (Amherst, MA: International Conference on Learning Representations). Available online at: https://openreview.net/

[B23] Hua X. Wang L. (2018). “Neural argument generation augmented with externally retrieved evidence,” in Proceedings of the 56th annual meeting of the association for computational linguistics (volume 1: long papers) (Melbourne, VIC: Association for Computational Linguistics), 219–230. doi: 10.18653/v1/P18-1021

[B24] Katsaros M. Yang K. Fratamico L. (2022). “Reconsidering tweets: intervening during tweet creation decreases offensive content,” in Proceedings of the international AAAI conference on web and social media, vol. 16 (Palo Alto, CA: AAAI Press), 477–487.

[B25] Kim Z. M. Du W. Raheja V. Kumar D. Kang D. (2022). “Improving iterative text revision by learning where to edit from other revision tasks,” in Proceedings of the 2022 conference on empirical methods in natural language processing, eds. Y. Goldberg, Z. Kozareva, and Y. Zhang (Abu Dhabi: Association for Computational Linguistics), 9986–9999. doi: 10.18653/v1/2022.emnlp-main.678

[B26] Laugier L. Pavlopoulos J. Sorensen J. Dixon L. (2021). “Civil rephrases of toxic texts with self-supervised transformers,” in Proceedings of the 16th conference of the european chapter of the association for computational linguistics: main volume (Stroudsburg, PA: Association for Computational Linguistics), 1442–1461. doi: 10.18653/v1/2021.eacl-main.124

[B27] Lawrence J. Reed C. (2019). Argument mining: a survey. Comput. Linguist. 45, 765–818. doi: 10.1162/coli_a_00364

[B28] Li Y. Wan X. (2026). “Edit-aware reward modeling for Chinese grammatical error correction,” in Proceedings of the 64th annual meeting of the association for computational linguistics (volume 1: long papers) (San Diego, CA: Association for Computational Linguistics), 40945–40957. doi: 10.18653/v1/2026.acl-long.1900

[B29] Li Y. Zhang P. Gu H. Lu T. Qiao S. Shu Y. . (2025). DeMod: a holistic tool with explainable detection and personalized modification for toxicity censorship. Proc. ACM Hum.-Comput. Interact. 9:CSCW061. doi: 10.1145/3710959

[B30] Liang J. Yang H. Gao S. Quan X. (2025). “Edit-wise preference optimization for grammatical error correction,” in Proceedings of the 31st international conference on computational linguistics (Abu Dhabi: Association for Computational Linguistics), 3401–3414.

[B31] Logacheva V. Dementieva D. Ustyantsev S. Moskovskiy D. Dale D. Krotova I. . (2022). “ParaDetox: detoxification with parallel data,” in Proceedings of the 60th annual meeting of the association for computational linguistics (volume 1: long papers) (Dublin: Association for Computational Linguistics), 6804–6818. doi: 10.18653/v1/2022.acl-long.469

[B32] Mller A. G. Romero D. M. Jurgens D. Aiello L. M. (2026). The impact of generative AI on social media: an experimental study. Sci. Rep. 16:9376. doi: 10.1038/s41598-026-40110-8 41699054 PMC13002914

[B33] Ouyang L. Wu J. Jiang X. Almeida D. Wainwright C. Mishkin P. . (2022). Training language models to follow instructions with human feedback. Adv. Neural Inform. Process. Syst. 35, 27730–27744. doi: 10.52202/068431-2011

[B34] Radford A. Wu J. Child R. Luan D. Amodei D. Sutskever I. (2019). Language Models are Unsupervised Multitask Learners. Technical report. San Francisco, CA: OpenAI.

[B35] Raheja V. Kumar D. Koo R. Kang D. (2023). “CoEdIT: text editing by task-specific instruction tuning,” in Findings of the association for computational linguistics: EMNLP 2023 (Singapore: Association for Computational Linguistics), 5274–5291. doi: 10.18653/v1/2023.findings-emnlp.350

[B36] Schechter Vera H. Dua S. Zhang B. Salz D. Mullins R. Panyam S. R. (2025). EmbeddingGemma: Powerful and Lightweight Text Representations. Technical report, Google DeepMind. arXiv:2509.20354. Available online at: https://arxiv.org/abs/2509.20354

[B37] Schulman J. Wolski F. Dhariwal P. Radford A. Klimov O. (2017). Proximal Policy Optimization Algorithms. Technical report, OpenAI. arXiv:1707.06347. Available online at: https://arxiv.org/abs/1707.06347

[B38] Shao Z. Wang P. Zhu Q. Xu R. Song J. Bi X. . (2024). DeepSeekMath: pushing the limits of mathematical reasoning in open language models. Technical report, DeepSeek-AI. arXiv:2402.03300. Available online at: https://arxiv.org/abs/2402.03300

[B39] Shu L. Luo L. Hoskere J. Zhu Y. Liu Y. Tong S. . (2024). “RewriteLM: an instruction-tuned large language model for text rewriting,” in Proceedings of the AAAI conference on artificial intelligence, vol. 38 (Washington, DC: AAAI Press), 18970–18980.

[B40] Singhal M. Ling C. Paudel P. Thota P. Kumarswamy N. Stringhini G. . (2023). “Sok: content moderation in social media, from guidelines to enforcement, and research to practice,” in 2023 IEEE 8th European symposium on security and privacy (EuroS&P) (Piscataway, NJ: IEEE), 868–895.

[B41] Skitalinskaya G. Spliethöver M. Wachsmuth H. (2023). “Claim optimization in computational argumentation,” in Proceedings of the 16th international natural language generation conference (Prague: Association for Computational Linguistics), 134–152. doi: 10.18653/v1/2023.inlg-main.10

[B42] Sreeraj S. Ananth M. Pradeep G. Barakkath Nisha U. (2025). Advanced cyberbullying detection integrating Pytesseract, Demoji and Bert for comprehensive textual and visual content analysis. Grenze Int. J. Eng. Technol. 11.

[B43] Stab C. Gurevych I. (2017). Parsing argumentation structures in persuasive essays. Comput. Linguist. 43, 619–659. doi: 10.1162/COLI_a_00295

[B44] Stahl M. Michel N. Kilsbach S. Schmidtke J. Rezat S. Wachsmuth H. (2024). “A school student essay corpus for analyzing interactions of argumentative structure and quality,” in Proceedings of the 2024 conference of the north american chapter of the association for computational linguistics: human language technologies (volume 1: long papers) (Mexico City: Association for Computational Linguistics), 2661–2674. doi: 10.18653/v1/2024.naacl-long.145

[B45] Stiennon N. Ouyang L. Wu J. Ziegler D. Lowe R. Voss C. . (2020). Learning to summarize with human feedback. Adv. Neural Inform. Process. Syst. 33, 3008–3021.

[B46] Syed S. Al Khatib K. Alshomary M. Wachsmuth H. Potthast M. (2021). “Generating informative conclusions for argumentative texts,” in Findings of the association for computational linguistics: ACL-IJCNLP 2021 (Stroudsburg, PA: Association for Computational Linguistics), 3482–3493. doi: 10.18653/v1/2021.findings-acl.306

[B47] Taori R. Gulrajani I. Zhang T. Dubois Y. Li X. Guestrin C. . (2023). Stanford Alpaca: An Instruction-Following LLaMA Model. Available online at: https://github.com/tatsu-lab/stanford_alpaca (Accessed April 23, 2026).

[B48] Toledo A. Gretz S. Cohen-Karlik E. Friedman R. Venezian E. Lahav D. . (2019). “Automatic argument quality assessment - new datasets and methods,” in Proceedings of the 2019 conference on empirical methods in natural language processing and the 9th international joint conference on natural language processing (EMNLP-IJCNLP) (Hong Kong: Association for Computational Linguistics), 5625–5635. doi: 10.18653/v1/D19-1564

[B49] Vikas J. Sanmuga Priya M. Barakkath Nisha U. (2025). Harnessing PCA - refined GloVe features with RoBERTaNET transformer learning for robust cyberbullying and cyberstalking detection. Grenze Int. J. Eng. Technol. 11.

[B50] Wachsmuth H. Naderi N. Hou Y. Bilu Y. Prabhakaran V. Thijm T. A. . (2017). “Computational argumentation quality assessment in natural language,” in Proceedings of the 15th conference of the European chapter of the association for computational linguistics: volume 1, long papers (Valencia: Association for Computational Linguistics), 176-187.

[B51] Wachsmuth H. Werner T. (2020).”Intrinsic quality assessment of arguments,” in *Proceedings of the 28th international conference on computational linguistics* (Barcelona: International Committee on Computational Linguistics), 6739–6745. doi: 10.18653/v1/2020.coling-main.592

[B52] Warner B. Chaffin A. Clavié B. Weller O. Hallström O. Taghadouini S. . (2025). “Smarter, better, faster, longer: a modern bidirectional encoder for fast, memory efficient, and long context finetuning and inference,” in Proceedings of the 63rd annual meeting of the association for computational linguistics (volume 1: long papers) (Vienna: Association for Computational Linguistics), 2526–2547. doi: 10.18653/v1/2025.acl-long.127

[B53] Wu C. Ren X. Luo F. Sun X. (2019). “A hierarchical reinforced sequence operation method for unsupervised text style transfer,” in Proceedings of the 57th annual meeting of the association for computational linguistics (Florence: Association for Computational Linguistics), 4873–4883. doi: 10.18653/v1/P19-1482

[B54] Wulczyn E. Thain N. Dixon L. (2017). “Ex machina: personal attacks seen at scale,” in Proceedings of the 26th international conference on world wide web, WWW '17 (Geneva: International World Wide Web Conferences Steering Committee), 1391–1399. doi: 10.1145/3038912.3052591

[B55] Zhang T. Kishore V. Wu F. Weinberger K. Q. Artzi Y. (2020). “BERTscore: evaluating text generation with BERT,” in International conference on learning representations. (Amherst, MA: International Conference on Learning Representations). Available online at: https://openreview.net/

[B56] Ziegenbein T. Skitalinskaya G. Bayat Makou A. Wachsmuth H. (2024). “LLM-based rewriting of inappropriate argumentation using reinforcement learning from machine feedback,” in Proceedings of the 62nd annual meeting of the association for computational linguistics (volume 1: long papers) (Bangkok: Association for Computational Linguistics), 4455–4476. doi: 10.18653/v1/2024.acl-long.244

[B57] Ziegenbein T. Stahl M. Wachsmuth H. (2026). “Teaching LLMs human-like editing of inappropriate argumentation via reinforcement learning,” in Proceedings of the 64th annual meeting of the Association for Computational Linguistics (Volume 1: Long Papers), San Diego, CA, USA, July 2026 (Stroudsburg, PA: Association for Computational Linguistics), 38616–38637. arXiv:2604.12770.

[B58] Ziegenbein T. Syed S. Lange F. Potthast M. Wachsmuth H. (2023). “Modeling appropriate language in argumentation,” in Proceedings of the 61st annual meeting of the association for computational linguistics (volume 1: long papers) (Toronto, ON: Association for Computational Linguistics), 4344–4363. doi: 10.18653/v1/2023.acl-long.238

