# Supplementary material for: A unified evaluation framework for revising inappropriate arguments with edit-level guidance
Source: Front Artif Intell. 2026 Oct 30;9:1869755. doi: 10.3389/frai.2026.1869755 (PMC13759734; doi:10.3389/frai.2026.1869755)
Supplement: Supplementary file 1 [file Table_1.pdf]

Table S1. Supplementary Table S1. Robustness of the selection model f_{sel}: standardized coefficients (*KoE* is the reference category), standard errors in parentheses; $n = 17{,}906$ candidate edit suggestions from 200 arguments and five annotators. *Clustered*: argument-clustered standard errors, as reported in the main manuscript's user study section. *(i)*: annotator fixed effects with argument-clustered standard errors. *(ii)*: two-way cluster-robust standard errors (argument × annotator), imprecise as they rest on only five annotator clusters. *(iii)*: crossed random intercepts for argument and annotator, estimated by variational Bayes; posterior means and posterior standard deviations, for which no p-values are defined. *(iv)*: conditional logit within groups of edit suggestions competing for the same source span, informed by 1,529 groups with 3,135 selections. $^{*}p < .05$, $^{**}p < .01$, $^{***}p < .001$.

	**Clustered**	**(i) Annot. FE**	**(ii) Two-way**	**(iii) Crossed RI**	**(iv) Cond. logit**
KiE	0.057^{*} (0.026)	0.057^{*} (0.026)	0.057 (0.056)	0.090 (0.018)	0.141^{***} (0.028)
Rep	0.162^{***} (0.026)	0.162^{***} (0.026)	0.162^{**} (0.061)	0.185 (0.017)	0.180^{***} (0.025)
Del	0.044^{*} (0.021)	0.044^{*} (0.022)	0.044 (0.038)	0.041 (0.018)	0.069^{**} (0.022)
Add	−0.056 (0.032)	−0.056 (0.032)	−0.056 (0.125)	−0.039 (0.019)	0.014 (0.030)

Table S2. Supplementary Table S2. Robustness of the appropriateness model f_{app}: standardized coefficients (*KoE* is the reference category), standard errors in parentheses; $n = 3{,}663$ selected edit suggestions from 200 arguments and five annotators. Specifications as in Table S1. $^{*}p < .05$, $^{**}p < .01$, $^{***}p < .001$.

	**Clustered**	**(i) Annot. FE**	**(ii) Two-way**	**(iii) Crossed RI**
KiE	−0.050 (0.058)	−0.045 (0.065)	−0.050 (0.075)	−0.088 (0.054)
Rep	0.324^{***} (0.071)	0.376^{***} (0.084)	0.324^{***} (0.088)	0.264 (0.063)
Del	0.100 (0.059)	0.081 (0.068)	0.100 (0.066)	0.132 (0.055)
Add	0.194^{*} (0.076)	0.200^{*} (0.093)	0.194^{*} (0.089)	0.347 (0.060)

Table S3. Supplementary Table S3. Edit intent prediction with uncertainty estimates: point estimates with percentile 95% confidence intervals from $B = 2{,}000$ bootstrap resamples of the 354 test edit suggestions. All predictions are obtained once per system, with greedy decoding (temperature 0) for the prompted models, so no sampling variation at generation time enters the comparison; the *Random* baseline is redrawn on every resample. The best value per column is marked bold.

**Method**	**Macro F_1**	**Micro F_1**	**Jaccard**
Random	0.454 $[0.429, 0.475]$	0.388 $[0.355, 0.418]$	0.267 $[0.236, 0.293]$
Majority	0.421 $[0.410, 0.431]$	0.535 $[0.490, 0.581]$	0.524 $[0.477, 0.572]$
Delta Replace	0.439 $[0.416, 0.462]$	0.304 $[0.268, 0.338]$	0.181 $[0.155, 0.207]$
Delta Mask	0.467 $[0.444, 0.487]$	0.352 $[0.319, 0.382]$	0.209 $[0.183, 0.234]$
Gemini 2.5	0.554 $[0.532, 0.576]$	**0.639** $[0.607, 0.669]$	**0.568** $[0.531, 0.605]$
+ Argument	0.532 $[0.511, 0.553]$	0.602 $[0.567, 0.636]$	0.524 $[0.484, 0.561]$
ModernBERT	0.616 $[0.592, 0.639]$	0.616 $[0.579, 0.651]$	0.545 $[0.504, 0.585]$
+ Argument	**0.652** $[0.615, 0.688]$	0.621 $[0.585, 0.657]$	0.544 $[0.502, 0.586]$

Table S4. Supplementary Table S4. Paired system comparisons: differences $A - B$ with percentile 95% confidence intervals, computed on shared bootstrap resample indices so that the comparisons are paired. Significance is based on two-sided bootstrap p-values, i.e. twice the smaller tail proportion of the difference distribution. *MB* abbreviates *ModernBERT*, *Arg* the variant with argument context. $^{*}p < .05$, $^{**}p < .01$, $^{***}p < .001$.

Comparison	Macro F_1	Micro F_1	Jaccard
Gemini − Random	$+0.101^{***}$ [+0.069, +0.134]	$+0.251^{***}$ [+0.211, +0.296]	$+0.301^{***}$ [+0.257, +0.348]
Gemini − Majority	$+0.134^{***}$ [+0.112, +0.156]	$+0.104^{***}$ [+0.065, +0.142]	$+0.044^{*}$ [+0.008, +0.082]
Gemini − Delta Replace	$+0.115^{***}$ [+0.084, +0.147]	$+0.334^{***}$ [+0.290, +0.379]	$+0.387^{***}$ [+0.343, +0.430]
Gemini − Delta Mask	$+0.088^{***}$ [+0.058, +0.118]	$+0.287^{***}$ [+0.245, +0.333]	$+0.359^{***}$ [+0.315, +0.402]
MB − Gemini	$+0.061^{***}$ [+0.030, +0.091]	−0.023 [−0.071, +0.023]	−0.023 [−0.073, +0.026]
MB+Arg − Gemini+Arg	$+0.120^{***}$ [+0.077, +0.161]	+0.019 [−0.027, +0.065]	+0.021 [−0.029, +0.071]
Gemini+Arg − Gemini	−0.022 [−0.043, +0.000]	-0.037^{**} [−0.062, −0.010]	-0.044^{**} [−0.074, −0.015]
MB+Arg − MB	$+0.036^{*}$ [+0.001, +0.070]	+0.006 [−0.028, +0.039]	−0.001 [−0.037, +0.035]

Table S5. Supplementary Table S5. Edit-level (App_e) and argument-level (App_a) appropriateness metrics broken down by inappropriateness dimension. Cells show absolute counts with percentages in parentheses.

Approach	Edit-Level (App_e)			Argument-Level (App_a)		
	Toxic Emotions	Missing Commitment	Missing Intelligibility	Toxic Emotions	Missing Commitment	Missing Intelligibility
**RL — Rewriting**						
Alpaca	20 (10.0%)	40 (19.9%)	170 (84.6%)	10 (8.4%)	10 (21.3%)	15 (27.8%)
+ PPO_{app}	<u>24 (35.3%)</u>	<u>42 (61.8%)</u>	35 (51.5%)	6 (5.0%)	<u>8 (17.0%)</u>	6 (11.1%)
+ $\text{PPO}_{app>sim}$	18 (26.9%)	33 (49.3%)	37 (55.2%)	<u>10 (8.4%)</u>	3 (6.4%)	9 (16.7%)
+ $\text{PPO}_{app=sim}$	18 (15.0%)	37 (30.8%)	94 (78.3%)	10 (8.4%)	6 (12.8%)	<u>11 (20.4%)</u>
+ $\text{PPO}_{app<sim}$	10 (5.7%)	14 (8.0%)	164 (93.7%)	7 (5.9%)	8 (17.0%)	14 (25.9%)
**RL — Editing**						
Llama	65 (11.8%)	130 (23.6%)	451 (81.7%)	16 (13.4%)	13 (27.7%)	20 (37.0%)
+ GRPO_{app}	32 (23.0%)	72 (51.8%)	93 (66.9%)	9 (7.6%)	6 (12.8%)	7 (13.0%)
+ GRPO_{con}	69 (16.4%)	177 (42.1%)	308 (73.3%)	17 (14.3%)	15 (31.9%)	25 (46.3%)
+ GRPO_{flu}	16 (66.7%)	19 (79.2%)	7 (29.2%)	2 (1.7%)	2 (4.3%)	1 (1.9%)
+ GRPO_{sim}	54 (7.9%)	133 (19.4%)	597 (87.3%)	21 (17.6%)	13 (27.7%)	22 (40.7%)
+ $\text{GRPO}_{\neg con}$	72 (22.4%)	117 (36.4%)	225 (70.1%)	19 (16.0%)	11 (23.4%)	11 (20.4%)
+ $\text{GRPO}_{\neg flu}$	52 (6.1%)	171 (20.0%)	<u>754 (88.2%)</u>	24 (20.2%)	17 (36.2%)	<u>26 (48.1%)</u>
+ $\text{GRPO}_{\neg sim}$	63 (18.6%)	123 (36.3%)	242 (71.4%)	15 (12.6%)	11 (23.4%)	17 (31.5%)
+ GRPO_{full}	<u>88 (12.8%)</u>	<u>184 (26.7%)</u>	558 (81.0%)	<u>25 (21.0%)</u>	<u>16 (34.0%)</u>	<u>21 (38.9%)</u>
**Prompting**						
Gemini 2.5	59 (9.9%)	138 (23.1%)	509 (85.1%)	14 (11.8%)	11 (23.4%)	21 (38.9%)
GPT-5	**94 (6.7%)**	**233 (16.7%)**	**1249 (89.4%)**	**27 (22.7%)**	**17 (36.2%)**	**25 (46.3%)**
**Supervised**						
DELITERATER	10 (4.6%)	8 (3.7%)	209 (95.9%)	15 (12.6%)	9 (19.1%)	17 (31.5%)
CoEdIT	0 (0.0%)	1 (0.3%)	<u>376 (99.7%)</u>	15 (12.6%)	12 (25.5%)	16 (29.6%)
